# Reduced Graphene Oxides as Carbocatalysts in Acceptorless Dehydrogenation of *N*-Heterocycles

**DOI:** 10.1021/acscatal.1c04649

**Published:** 2021-11-23

**Authors:** Andrés Mollar-Cuni, David Ventura-Espinosa, Santiago Martín, Hermenegildo García, Jose A. Mata

**Affiliations:** † Institute of Advanced Materials (INAM), Centro de Innovación en Química Avanzada (ORFEO−CINQA), Universitat Jaume I, Avda. Sos Baynat s/n, 12006, Castellón, Spain; ‡ Instituto de Nanociencia y Materiales de Aragón (INMA), CSIC-Universidad de Zaragoza, Zaragoza 50009, Spain; § Departamento de Química Física, Universidad de Zaragoza, 50009 Zaragoza, Spain; ⊥ Laboratorio de Microscopias Avanzadas (LMA), Universidad de Zaragoza, Edificio I+D+i, 50018 Zaragoza, Spain; ∥ Instituto de Tecnología Química, Consejo Superior de Investigaciones Científicas-Universitat Politècnica de València, Avda. Los Naranjos s/n, 46022, Valencia, Spain

**Keywords:** heterogeneous catalysis, carbocatalysis, reduced graphene oxide, *N*-heterocycles, dehydrogenation, graphene active sites

## Abstract

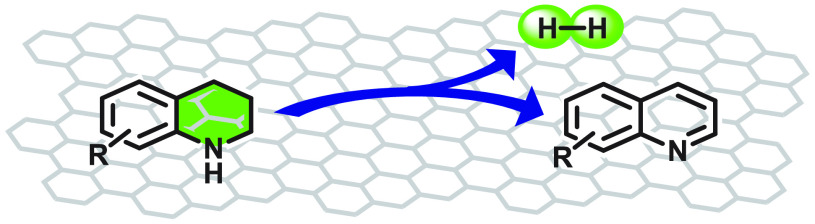

The catalytic properties of graphene-derived materials are evaluated in acceptorless dehydrogenation of *N*-heterocycles. Among them, reduced graphene oxides (rGOs) are active (quantitative yields in 23 h) under mild conditions (130 °C) and act as efficient heterogeneous carbocatalysts. rGO exhibits reusability and stability at least during eight consecutive runs. Mechanistic investigations supported by experimental evidence (i.e., organic molecules as model compounds, purposely addition of metal impurities and selective functional group masking experiments) suggest a preferential contribution of ketone carbonyl groups as active sites for this transformation.

Metal-free catalysts might play an important role in the design of sustainable and environmentally friendly chemical transformations.^1−4^ The actual dependence of catalysis on metals, often precious and rare, is no longer sustainable, because of the cost, limited abundance, and depletion of metal sources. Development of metal-free catalysts based on abundant elements is a promising area of research. Organocatalysis is a well-established field in which well-defined active sites are present in organic molecules.^5−7^ Parallel to this, the use of heterogeneous carbonaceous materials is gaining interest, but the area is in its earlier development, in terms of understanding the reaction mechanisms, architecture of the active sites, and engineering of materials with single sites.^8−10^ In recent years, we have witnessed a rapid development of graphene-type materials as catalysts.^11,12^ Defective graphenes have shown catalytic activity in oxidation,^13,14^ reduction,^15,16^ and coupling reactions.^17,18^

In parallel, the hydrogenation and dehydrogenation of *N*-heterocycles is an important reaction from the synthetic viewpoint. *N*-heterocycles are found in many highly added-value organic compounds. They are also considered as potential liquid organic hydrogen carriers (LOHCs) for hydrogen storage and release.^19−21^ One advantage of *N*-heterocycles as LOHCs, compared to cycloalkanes, is the reduction of dehydrogenation enthalpy facilitating hydrogen release.^22−24^ The success of H_2_ storage in organic compounds is dependent on the development of efficient, stable, and affordable catalysts.^25−27^ However, to the best of our knowledge, acceptorless dehydrogenation (ADH) of tetrahydroquinolines using metal-free catalysts has not been reported. Previous examples describe the use of advanced nanostructures (e.g., nanodiamons) or activated mesoporous carbons in acceptorless gas-phase dehydrogenation of alkanes at high temperatures (>500 °C).^28−30^ Alternatively, graphene oxide (GO) or activated carbon (AC) have been used in the oxidative dehydrogenation of *N*-heterocycles with the concomitant formation of water or hydrogen peroxide (Figure 1).^31−35^ In this manuscript, we describe the activity of reduced graphene oxides (rGOs) as efficient and reusable carbocatalysts for the ADH of tetrahydroquinolines with the production of molecular hydrogen, a key reaction for on-board hydrogen release. Our research provides a general scope of the metal-free ADH carbocatalysts as well as evidence of the active sites responsible for this transformation.

**Figure 1:**
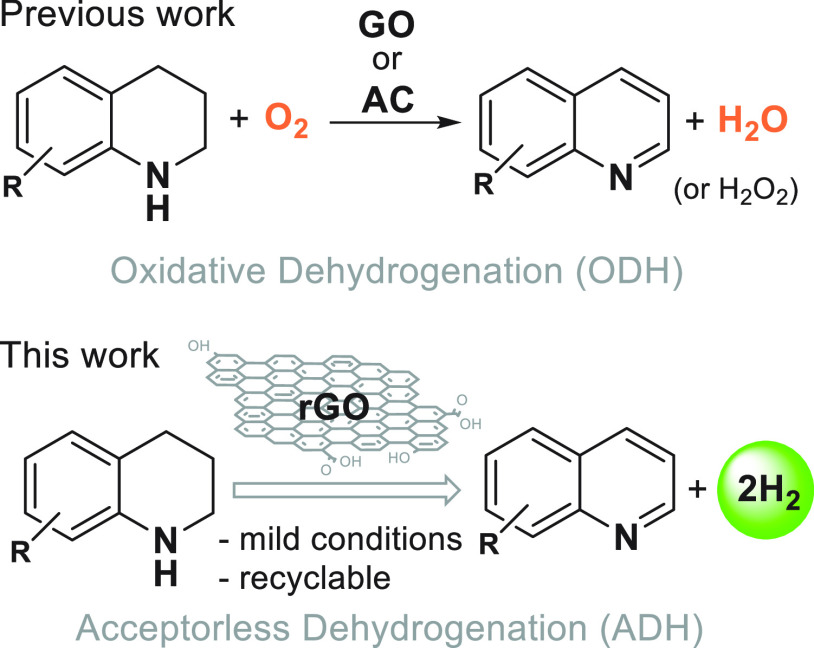
Differences between oxidative and acceptorless dehydrogenation of *N*-heterocycles. The latter is important for H_2_ storage. In ADH, H_2_ gas is released while in ODH hydrogen is transferred to oxygen and released as H_2_O. [Legend: GO, graphene oxide; AC, activated carbon, and rGO, reduced graphene oxide.]

The performance of rGO as carbocatalyst in ADH of *N*-heterocycles was first evaluated using 1,2,3,4-tetrahydroquinoline (THQ, **1H**) as a model substrate under oxygen-free conditions (see Table 1, as well as Figure S1 in the Supporting Information (SI)). Our first concern was to determine whether rGO was acting as a true carbocatalyst or as a stoichiometric reagent.^36^ First of all, we confirmed that dehydrogenation of THQ did not occur without rGO (Table 1, entry 1). In the presence of rGO, quinoline is obtained under different solvents and reaction conditions, indicating that the process is general. ADH of **1H** afforded quinoline (**1D**) with the concomitant release of two molecules of H_2_. Hydrogen was qualitatively analyzed using a microGC. The ADH of N-heterocycles is an endergonic reaction, in contrast to the ODH. The driving force of ADH is the removal of hydrogen from the reaction media. In fact, when the dehydrogenation reaction is performed in a closed system, no product formation occurred (Table 1, entry 8). Then, the influence of catalyst loading in the dehydrogenation of 8-methoxytetrahydroquinoline (**7H**) was assessed (Figure S1). The apparent reaction rates are dependent on the amount of rGO, and the reaction profiles suggest a catalytic nature for the reaction. For instance, lowering the catalyst loading to 5 mg considerably decreased the apparent reaction rate, but still a good yield (68%) could be obtained after 23 h. The selectivity and the presence of other reaction products was evaluated through analogous experiments using deuterated *o*-DCB and by analyzing the reaction mixture without any purification process (Figures S2–S4 in the SI). By monitoring the reaction progress by ^1^H NMR spectroscopy, it could be seen how the starting material (**3H**) is converted to the corresponding dehydrogenated product (**3D**) without the formation of other products. This confirmed the high selectivity of ADH of THQs toward quinolines using rGO as a carbocatalyst.

**Table 1:**
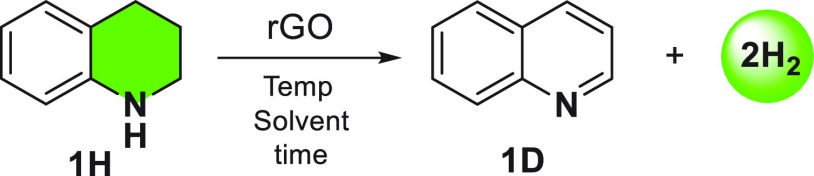
Catalytic Activity of rGO in ADH of 1,2,3,4-Tetrahydroquinoline (**1H**) under Various Reaction Conditions^a^

entry	solvent	temperature, *T* (°C)	conversion (%)	yield (%)
1^b^	*o*-DCB	130	5	1
2	*o*-DCB	130	91	85
3	*o*-DCB	110	78	67
4	DMF	130	74	58
5	toluene	110	14	9
6	*n*-decane	130	81	64
7	DIPB	130	79	58
8^d^	*o*-DCB	130	10	n.d.

a Reaction conditions: 1,2,3,4-tetrahydroquinoline (0.15 mmol), rGO (15 mg), solvent (1 mL) for 23 h. Evolution of starting material (conversion) obtained by GC/FID using 1,3,5-trimethoxybenzene as internal standard and product formation (yield) obtained by ^1^H NMR analysis.

b Without rGO.

c Legend: *o*-DCB: *ortho*-dichlorobenzene; DMF: *N*,*N*-dimethylformamide: and DIPB, 1,3-diisopropylbenzene.

d Closed system.

Next, we investigated the scope and limitations of *N*-heterocycle dehydrogenation, using rGO as a carbocatalyst (see Table 2). The reactions were monitored by GC, and the activity was compared using apparent reaction rates. (See the SI for details.) rGO is an efficient carbocatalyst for a variety of substituted THQs and indolines. Methyl (**3H**) or phenyl (**5H**) substitution at the 2-position of THQ showed similar rates and afforded quantitative yields after 23 h. Introduction of different groups at the 6-position (**6H** and **7H**) does not influence the formation of quinolines, and similar reaction rates were obtained. A substantial limitation in ADH of THQ substituted at the 8-position was observed. The presence of a methyl (**2H**) or a phenyl (**8H**) reduced the yield of **2D** and **8D** to 56% and 50%, respectively, probably because of steric effects. The same procedure was used for dehydrogenation of tetrahydroisoquinoline (**10H**). Under these conditions, full conversion of **10H** was also obtained, but only 15% yield corresponds to isoquinoline (**10D**): the rest (∼70% yield) is the monodehydrogenated product. Monitoring of the temporal reaction profile indicates that double dehydrogenation of **10H** requires longer reaction times (Figure S12 in the SI). A fast reaction and quantitative conversion was obtained for tetrahydroquinoxaline (**4H**) containing two nitrogen groups (92% conversion over 8 h). This result is in agreement with previous theoretical calculations, revealing that the dehydrogenation is thermodynamically favored by increasing the number of N atoms in the *N*-heterocycles.^23^ An important limitation was found for tetrahydronaphthyridine (**9H**), while a good conversion of 76% was observed; in contrast, the product **9D** could only be obtained in a low yield (32%), because of product degradation. Further studies with five-membered ring *N*-heterocycles (indolines) showed faster reactions. For instance, indoline (**11H**) and 2-methylindoline (**12H**) were fully dehydrogenated to the corresponding indole (**11D**) or 2-methylindole (**12D**) within <10 h.

**Table 2:**
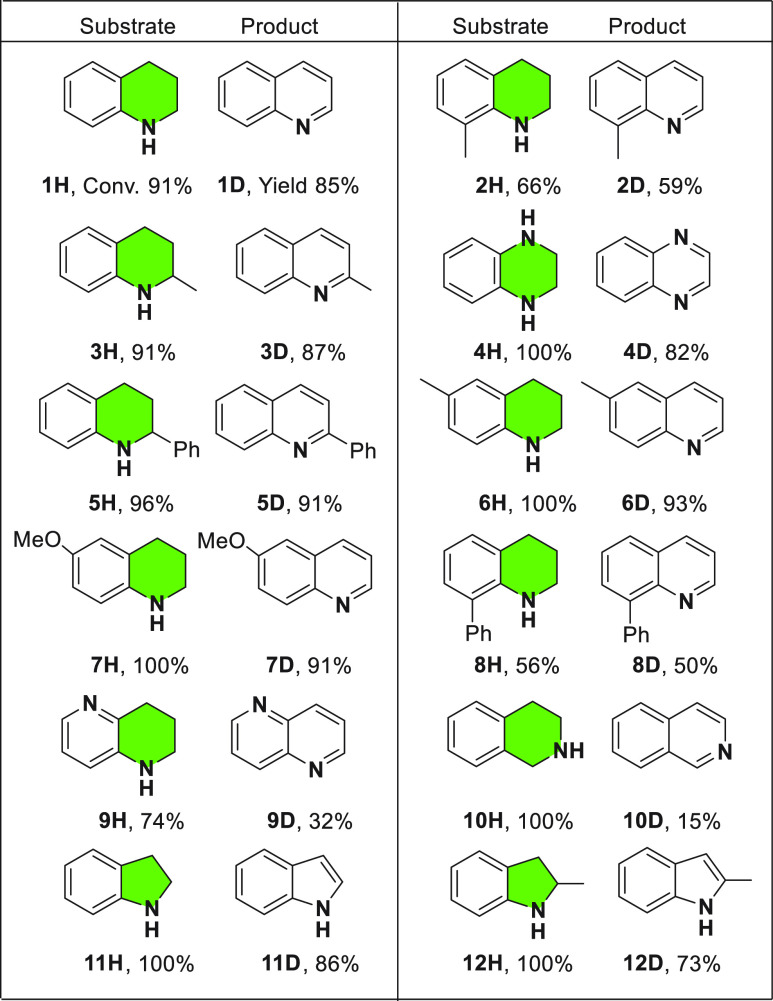
Scope of ADH of *N*-Heterocycles^a^

a Reaction conditions: Substrate (0.15 mmol), 15 mg of rGO, *o*-DCB (1 mL) at 130 °C for 23 h. The number under the starting material corresponds to conversion obtained by GC/FID and the number under the product corresponds to the isolated product yield after purification. See the SI for the reaction profiles and details. “H” denotes hydrogenated; “D” denotes “dehydrogenated”.

The performance of rGO as a carbocatalyst was further evaluated by recycling experiments. The temporal reaction profile provides valuable information of activity and stability. The rGO was removed from the solution after each run by decantation, washed with MeOH, and reused without any preactivation process. The data presented in Figure 2 are the average of two independent reactions. These results show some decrease in activity from run 1 to run 2 and then, from run 4 to run 6. However, the activity is recovered from runs 7 and 8. These fluctuations are due to the imperfect experimental working procedure with rGO, rather than a real tendency to deactivation. In any case, the catalytic activity of rGO is maintained for eight consecutive runs. Note that we have not observed any sign of catalyst deactivation. This fact suggests that rGO is a stable carbocatalyst. After the recycling experiments, the spent rGO was analyzed by high-resolution transmission electron microscopy (HRTEM), X-ray photoelectron spectroscopy (XPS), elemental analysis, and Raman spectroscopy to determine any changes in the morphology and composition (Figures S15–S17 in the SI). The only subtle difference is the presence of more wrinkles in the spent rGO carbocatalyst. We believe that the presence of these wrinkles is not directly related to the catalytic activity, but rather a consequence of the use of rGO in multiple experiments. XPS spectra of the C 1s and O 1s bands for the fresh and spent rGO show a similar peak shapes and at the same binding energies (see Figure S16). Deconvolution provides similar contributions of different bonding modes (C–C, C–N, C–O, C=O, and HO–C=O) for the fresh and spent rGO. The oxygen/carbon ratio for the fresh rGO sample was 0.12 and for the spent rGO (8 runs) was 0.13. These results confirm that, during the catalytic reaction, there is no increase or decrease in oxygen content. Elemental composition by combustion analyses confirmed that the oxygen/carbon ratio is maintained after exhaustive recycling (Table S3 in the SI). The Raman spectra provided the characteristic graphene pattern with D, G, and 2D bands. The relative intensity (*I*_D_/*I*_G_) of D and G bands is preserved after eight runs, indicating the high stability of rGO (see Figure S17). The available characterization data before and after the recycling experiment indicates that rGO is not altered during the dehydrogenation of *N*-heterocycles.

**Figure 2:**
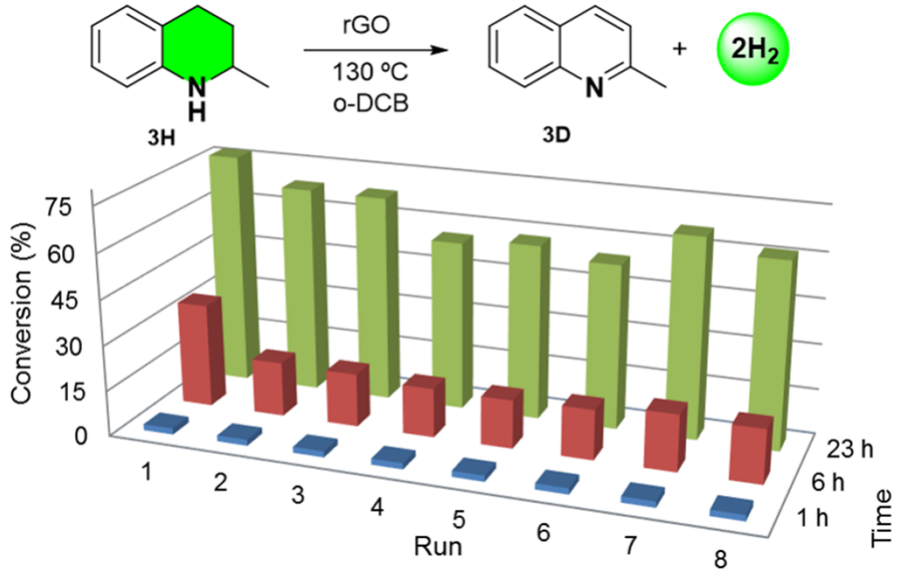
Activity of rGO as carbocatalyst for ADH of *N*-heterocycles. Reaction conditions: Tetrahydroquinaldine (**3H**, 0.3 mmol), catalyst loading (30 mg of rGO), with *o*-DCB (2.0 mL) as solvent at 130 °C. Conversion was determined by gas chromatography/flame ionization detection (GC/FID) and using 1,3,5-trimethoxybenzene as an internal standard.

Mechanistic studies of catalytic reactions using carbonaceous materials is a difficult task, because of the ill-defined nature of the active sites.^37^ To get information about the catalytic active sites of rGO, experiments were performed to address the influence of residual metals, the use of organic molecules as models of the active centers, and the selective masking of functional groups. We first assessed the catalytic activity of different graphene-related materials including rGO from different commercial suppliers. The results (Figure S25) showed that graphene (G) and graphite are not active in the dehydrogenation of *N*-heterocycles. GO is highly active at low conversions, but then it deactivates fast and, after a prolonged reaction time, only 63% yield is obtained. In contrast, all rGO materials used show a remarkable activity independently if they are obtained via thermal or chemical reduction. This observation indicates that the presence of vacancies or holes, which are more common in thermal rGO, or the presence of adventitious sites, such as N atoms in chemical reduction by hydrazine, are not necessary for the catalytic activity. The results derived from different carbonaceous materials indicate that the functional groups and the sp^2^ character of the basal plane of carbonaceous materials play a role as active sites in the catalytic reaction.

Then, we analyzed the potential catalytic activity of metal impurities in the carbonaceous materials. Carbonaceous materials are predominantly obtained from graphite that contains several metal impurities present at trace levels.^38−40^ These impurities could have an impact in the catalytic properties of graphene. In addition, the preparation of GO and rGO involves the use of oxidant/reducing agents, some of which can contain metals that may remain in the final samples, even at low concentrations. Common metal impurities found in rGO are iron and manganese. We evaluated the catalytic activity in dehydrogenation of *N*-heterocycles by adding known amounts of these metals in minute concentrations. The reaction progress in three parallel reactions—containing no added metal, with 0.5 mg of M^2+^, and with 1 mg of M^2+^—were monitored (see Figures S27 and S28 in the SI). The results show that the three curves overlapped for Mn^2+^ or Fe^2+^, suggesting that the catalytic effect induced by the presence of these metal ions at these concentrations is negligible.

We also used a series of organic molecules as models of the active sites to assess the role of different functional groups (−OH, −COOH, and C=O) in the ADH of *N*-heterocycles (see Figure S29 in the SI). The use of simple organic molecules for mimicking the role oxygenated groups at the surface of carbon materials is gaining interest, because of the relevant information provided.^41,42^ The results show that model molecules containing carboxylic acids and carbonyl groups somehow promote the dehydrogenation of *N*-heterocycles (see Table S5 in the SI). For instance, the yield of 2-methyltetraquinoline (**3D**) is 19% and 34% when using benzoic acid (A) and pyrene-4,5-dione (H), respectively. Other compounds, such as benzoquinone and phenol, also exhibit some activity. The results also suggested that the chemical environment of the functional groups is important, as we observed differences between functional groups attached to benzene or pyrene scaffolds. The activity of the model molecules suggests that carboxylic acids, hydroxy groups, and carbonyl groups present on rGO are potential catalytic active sites. We then used modified rGO samples in which certain functional groups have been selectively masked to provide further information on active sites. Carboxylic acids, ketone groups, and phenol groups of rGO were selectively masked using established procedures (see Scheme S1 in the SI).^43,44^ The catalytic activity of the parent rGO and the masked materials rGO^COOH^, rGO^CO^, and rGO^OH^ (where the superscript indicates the masked group) was evaluated in dehydrogenation of tetrahydroquinaldine (**3H**). The results reveal that samples without carboxylic groups or phenolic moieties exhibit lower apparent reaction rates than unprotected rGO. A more remarkable decrease in the dehydrogenation rates occurs upon masking the carbonyl groups (see Figure S32 in the SI). The results of masking experiments suggest that the carbonyl groups are the prevalent active sites that, together with the lesser activity of carboxylic and hydroxyl groups, are responsible for the performance of rGO.

Previous studies on the use of rGO as carbocatalyst have already claimed the role of carbonyl groups (quinone type) as active sites.^41,45,46^ Based on present experimental evidence, a plausible mechanism for the ADH of *N*-heterocycles is proposed in which the role of carbonyl groups is emphasized as previously observed in ODH (Figure 3).^47−49^ According to this proposal, dehydrogenation of the *N*-heterocycles would occur with the concomitant reduction of carbonyl groups that, in a subsequent step, would release molecular hydrogen.

**Figure 3:**
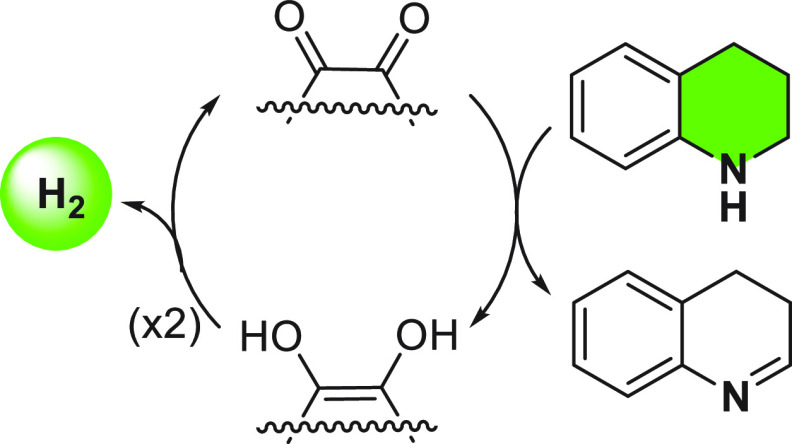
Plausible mechanism in the dehydrogenation of *N*-heterocycles, showing the role of carbonyl groups.

Graphene oxide (GO) is an efficient metal-free carbocatalyst for oxidative dehydrogenation (OHD) reactions. In this manuscript, reduced graphene oxide (rGO) behaves as a suitable carbocatalyst to promote, without metal assistance, a more challenging thermodynamically uphill reaction, namely, the acceptorless dehydrogenation (ADH) of *N*-heterocycles. In order the reaction to proceed, H_2_ must be removed from the system, while the catalyst must be very efficient in establishing equilibrium concentrations. rGOs of various origins are equally active as catalysts promoting dehydrogenation of a wide range of *N*-heterocycles. Available catalytic data indicate that possible metal impurities are not involved in the process and that the most likely active sites are quinone-like carbonylic groups. In this way, the present results represent a step forward toward the sustainability for on-board hydrogen release that could be applicable in massive scale without mining or depletion of limited metal resources.

## Supporting Information Available

Experimental details, synthetic procedures, mechanistic protocols, and full characterization of the carbonaceous materials (PDF)

## ABBREVIATIONS

rGO: reduced graphene oxide
GO: graphene oxide
ADH: acceptorless dehydrogenation
ODH: oxidative dehydrogenation
