# Supplementary material for: Reduced Graphene Oxides as Carbocatalysts in Acceptorless Dehydrogenation of N-Heterocycles
Source: ACS Catal. 2021 Nov 23;11(23):14688–93. doi: 10.1021/acscatal.1c04649 (PMC8711125; doi:10.1021/acscatal.1c04649)
Supplement: Supplementary file 1 — cs1c04649_si_001.pdf [file cs1c04649_si_001.pdf]

Reduced Graphene Oxides as Carbocatalysts in Acceptorless Dehydrogenation of N-Heterocycles

Andrés Mollar-Cuni,[a] *David Ventura-Espinosa,*[a] *Santiago Martín,*[b,c] *Hermenegildo García*[*d] *and Jose A. Mata*[*a]

[a]Institute of Advanced Materials (INAM), Centro de Innovación en Química Avanzada (ORFEO-CINQA), Universitat Jaume I, Avda. Sos Baynat s/n, 12006, Castellón (Spain). Tel: +34 964387516. E-mail: jmata@uji.es

[b]Instituto de Nanociencia y Materiales de Aragón (INMA), CSIC-Universidad de Zaragoza, Zaragoza 50009, Spain

[c]Departamento de Química Física, Universidad de Zaragoza, 50009, Zaragoza (Spain) and Laboratorio de Microscopias Avanzadas (LMA). Universidad de Zaragoza, Edificio I+D+i. 50018, Zaragoza, Spain

[d]Instituto de Tecnología Química, Consejo Superior de Investigaciones Científicas-Universitat Politècnica de València. Avda. Los Naranjos s/n, 46022, Valencia (Spain).

Table of Contents

S1 General procedures 3
Reagents and solvents 3
Instrumentation 3
Synthesis of graphenes 3
Catalytic dehydrogenation reactions 4
S2 Optimization of reaction conditions 5
S3 Detection of Molecular Hydrogen 5
S4 Influence of catalyst loading 6
S5 Selectivity: ^{1}H NMR spectroscopy reaction evolution in *o*-DCB-4d 6
S6 Scope and limitations: Reaction progress profiles 10
S7 Recycling experiment: Fresh and spent rGO characterization. 16
HRTEM microscopy 16
X-ray photoelectron spectroscopy (XPS) 17
Combustion Analysis 18
Raman spectroscopy 18
S8 Product characterization 19
S9 Catalytic properties of graphene materials in dehydrogenation of N-heterocycles 23
Comparative X-ray photoelectron spectroscopy (XPS) of chemical and thermal rGO. 24
S10 Influence of metal ions in the catalytic properties of rGO 25
S11 Functional groups: Model molecules used as carbocatalyst 26
S12 Dehydrogenation sequence of N-heterocycles 27
S13 Masking experiments 28
Masking of phenol type groups(rGO^{OH}) 28
Masking of carboxylic acid groups (rGO^{COOH}) 28
Masking of ketonic carbonyl groups (rGO^{CO}) 28
S14 Reported catalysts comparison in dehydrogenation of tetrahydroquinaldine dehydrogenation. 30
S15 References 31

S1 General procedures

Reagents and solvents

N-heterocycles were purchased from commercial suppliers and used without further purification. Anhydrous solvents were dried using a solvent purification system or purchased from commercial suppliers and stored over molecular sieves. Solvents were deoxygenated using the freeze-pump-thaw methodology and kept under an atmosphere of nitrogen.

Instrumentation

Nuclear magnetic resonance (**NMR**) spectra were recorded on Bruker spectrometers operating at 300 or 400 MHz (^{1}H NMR) and 75 or 100 MHz ($^{13}C\{^1H\}$ NMR), respectively, and referenced to $SiMe_4$ (δ in ppm and J in Hertz). NMR spectra were recorded at room temperature with the appropriate deuterated solvent. **Elemental Analysis** was carried out in a TruSpec Micro Series. High-resolution images of transmission electron microscopy (**HRTEM**) and high-angle annular dark-field (**HAADF-STEM**) images of the samples were obtained using a Jem-2100 LaB6 (JEOL) transmission electron microscope coupled with an INCA Energy TEM 200 (Oxford) energy dispersive X-Ray spectrometer (EDX) operating at 200 kV. Samples were prepared by drying a droplet of a MeOH dispersion on a carbon-coated copper grid. X-ray photoelectron spectra (**XPS**) were acquired on a Kratos AXIS ultra DLD spectrometer with a monochromatic Al Kα X-ray source (1486.6 eV) using a pass energy of 20 eV. To provide a precise energy calibration, the XPS binding energies were referenced to the C1s peak at 284.6 eV. Gas chromatography (**GC**) analyses were obtained on a shimadzu GC-2010 apparatus equipped with a FID detector, and using a Teknokroma column (TRB-5MS, 30 m x 0.25 mm x 0.25 µm).

Synthesis of graphenes

Reduced graphene oxide (**rGO**).

The employed reduced graphene oxide in dehydrogenation reactions of N-heterocycles was obtained from a commercial supplier (Graphenea). The same batch of rGO was used for all experiments in order to assure reproducibility. The rGO is produced by oxidation of graphite using the Hummer's method.[1] Then is chemically reduced using an organic reducing agent under basic conditions. Properties: Specific surface area 423 – 498 m^2/g; Electrical conductivity: 667 S/m; Density 0.06 – 0.09 g/cm^3.

Graphene oxide (**GO**)

Graphene oxide (GO) was prepared from graphite powder (natural, universal grade, 200 mesh, 99,9995%) by the Hummer's method.[1] Briefly, graphite is oxidized using potassium permanganate under strong acidic conditions (sodium nitrite and sulfuric acid). Exfoliation is performed using ultrasounds.

Thermal reduced graphene oxide (**rGO^t**)

The rGO^t was obtained by thermal reduction of GO. The GO (prepared by the Hummer's method) was heated in a furnace under inert atmosphere (Ar) at 200 ºC (5 ºC/min) for 4 h. Removal of functional groups produces a black material known as thermally reduced graphene oxide (rGO^t) that has been previously described.[2]

Graphene (**G**)

Graphene was prepared according to a literature procedure literature.[3] Commercial alginate, after purification by dissolution in aqueous solution and filtration under pressure, was pyrolyzed as powder on a ceramic crucible at 900 ºC (5 ºC/min) under an argon flow for 4h. The carbonaceous residue was submitted to sonication (250 W) for 30 min in water. The suspension was decanted, and the solid residue discarded. The suspension was freeze-dried and the graphenic material resuspended before using as a catalyst.

Catalytic dehydrogenation reactions

All catalytic experiments were carried out under an atmosphere of nitrogen using dry and deoxygenated solvents. In a typical procedure, the carbocatalyst was filled into a dry Schlenk flask (20 mL) connected to condenser containing a bubbler filled with mineral oil to exclude air from the reaction system and allowing the release of hydrogen gas. Solvent, substrate and 1,3,5-trimethoxybenzene as a standard were added under a nitrogen flux. The mixture was stirred at 130 ºC (bath temperature) for at least 23h. The reaction progress was monitored by gas chromatography taking aliquots at selected intervals.

S2 Optimization of reaction conditions

Table S1 Optimization of reaction conditions in dehydrogenation of 1,2,3,4-tetrahydroquinoline.

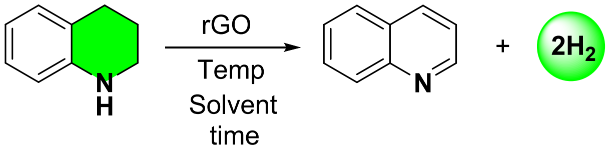

Entry	rGO (mg)	Substrate (mmol)	Solvent	T (^{0}C)	Time (h)	Conv. (%)	Yield (%)
1	-	0.1	*o*-DCB (2mL)	130	23	5	1
2	20	0.1	*o*-DCB (2mL)	130	23	100	85
3	10	0.3	*o*-DCB (2mL)	130	23	41	35
4	15	0.15	*o*-DCB (1mL)	130	23	91	85
5	15	0.15	*o*-DCB (1mL)	110	23	78	67
6	15	0.15	DMF(1mL)	130	23	74	58
7	15	0.15	toluene (1mL)	110	23	14	9
8	15	0.15	n-butanol (1mL)	120	23	18	12
9	15	0.15	n-decane (1mL)	130	23	81	64
10	15	0.15	DIPB (1mL)	130	23	79	58
11[a]	15	0.15	*o*-DCB (1mL)	130	23	10	n.d.
12[b]	15	0.15	*o*-DCB (1mL)	130	32	99	90
13[c]	15	0.15	*o*-DCB (1mL)	130	30	90	84

Reaction conditions: 1,2,3,4-tetrahydroquinoline (0.15 mmol), rGO, solvent for 23 h. Evolution of starting material (conversion) obtained by GC/FID using 1,3,5-trimethoxybenzene as an internal standard and product formation (yield) obtained by ^{1}H NMR analysis [a] Closed system. *o*-DCB, ortho dichlorobenzene; DMF, N,N-dimethylformamide; DIPB, 1,3-diisopropylbenzene. [b] Under aerobic conditions. [c] Under aerobic conditions in a closed system.

S3 Detection of Molecular Hydrogen

All glassware was carefully cleaned and rinsed with Milli-Q water prior to use. A 25 mL three necked round bottom flask was charged with 0.3 mmol of 1,2,3,4-tetrahydroquinaldine, 30 mg of rGO, 2 mL of *o*-DCB under N_2 atmosphere. The mixture was stirred at 130 ^{o}C at atmospheric pressure using an oil-bubbler. After 15 hours reaction, the gas evolved was collected with a syringe and directly injected into a quadrupole mass spectrometer equipment (Omnistar GSD 320 03 from PFEIFFER VACUUM). This

experiment qualitatively confirms the presence of molecular hydrogen in the acceptorless dehydrogenation of N-heterocycles.

S4 Influence of catalyst loading

The influence of catalyst loading in dehydrogenation of N-heterocycles was evaluated using 6-methoxytetraquinoline (**7H**) as model substrate. The catalytic reaction was monitored by gas chromatography using different amounts of rGO as carbocatalyst.

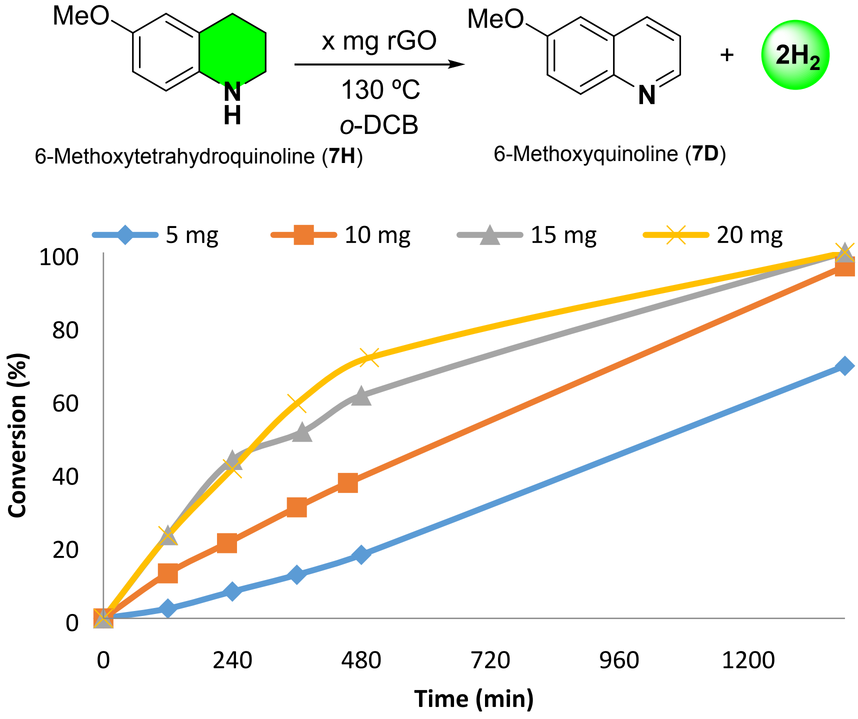

Figure S1 Reaction progress profiles: Influence of catalyst loading in dehydrogenation of **7H**. Conditions: Substrate (0.15 mmol), rGO (x mg), *o*-DCB (1 mL) at 130 ^{0}C. Conversion by GC/FID.

S5 Selectivity: ^{1}H NMR spectroscopy reaction evolution in *o*-DCB-4d

The selectivity in acceptorless dehydrogenation of THQs was evaluated by ^{1}H NMR spectroscopy using deuterated *o*-DCB. The catalytic experiments were carried out under the standard conditions described before (S1). Samples were taken at selected times and directly analyzed by ^{1}H NMR spectroscopy after microfiltration using a Pasteur pipette with a cotton plug to remove the solid catalyst.

Selectivity studies in the conversion of tetrahydroquinaldine (**3H**).

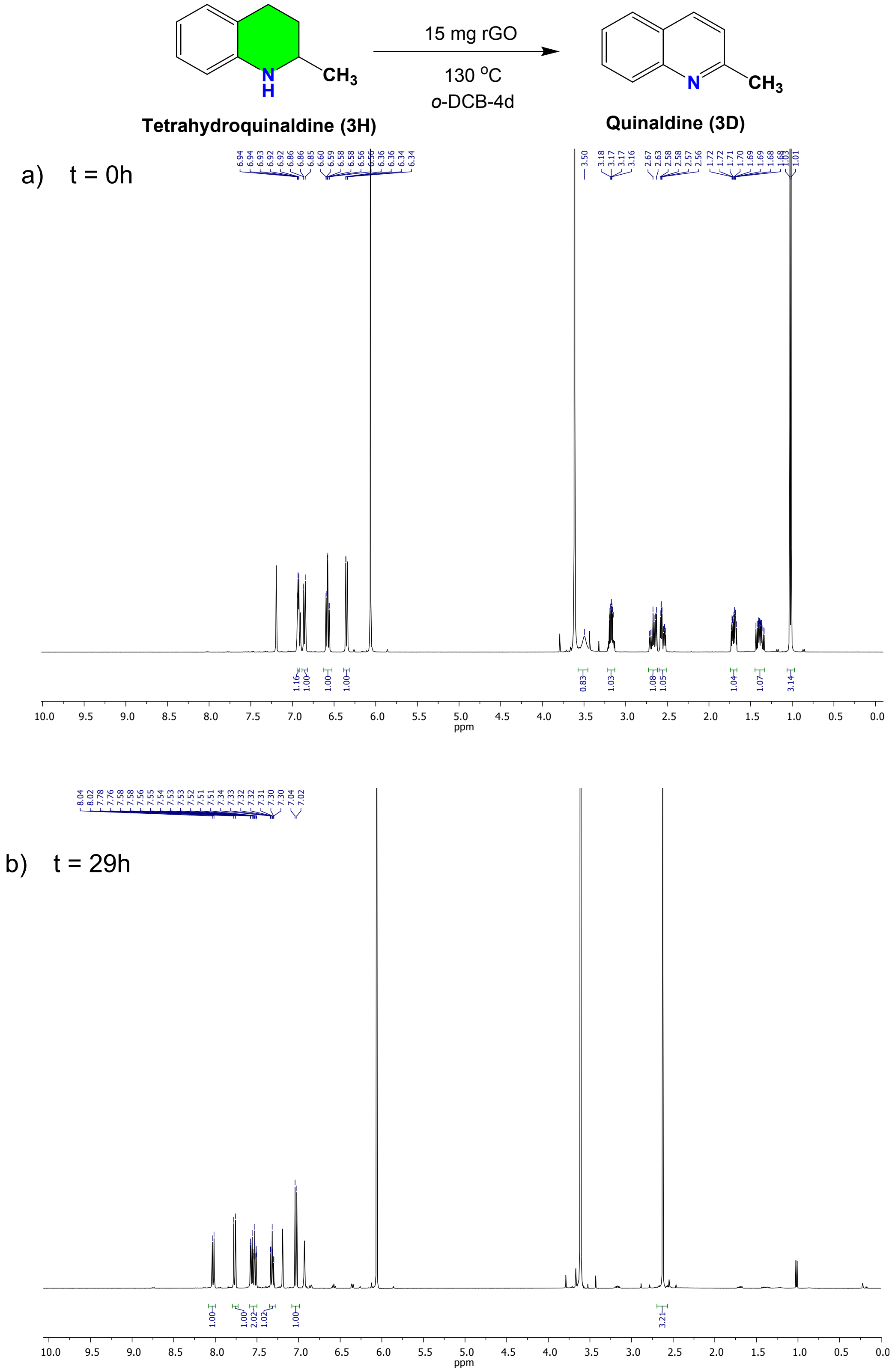

Figure S2 Acceptorless dehydrogenation of **3H**. Initial ^{1}H NMR spectrum (a) and after t = 29 h reaction (b) using 1,3,5-trimethoxybenzene (signals at 3.61 and 6.06 ppm) as an internal standard. Residual *o*-DCB solvent signals at 6.93 and 7.19 ppm.

Selectivity studies in the conversion of tetrahydroquinoxaline (**4H**).

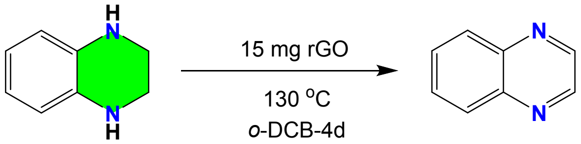

1,2,3,4-Tetrahydroquinoxaline (4H)

Quinoxaline (4D)

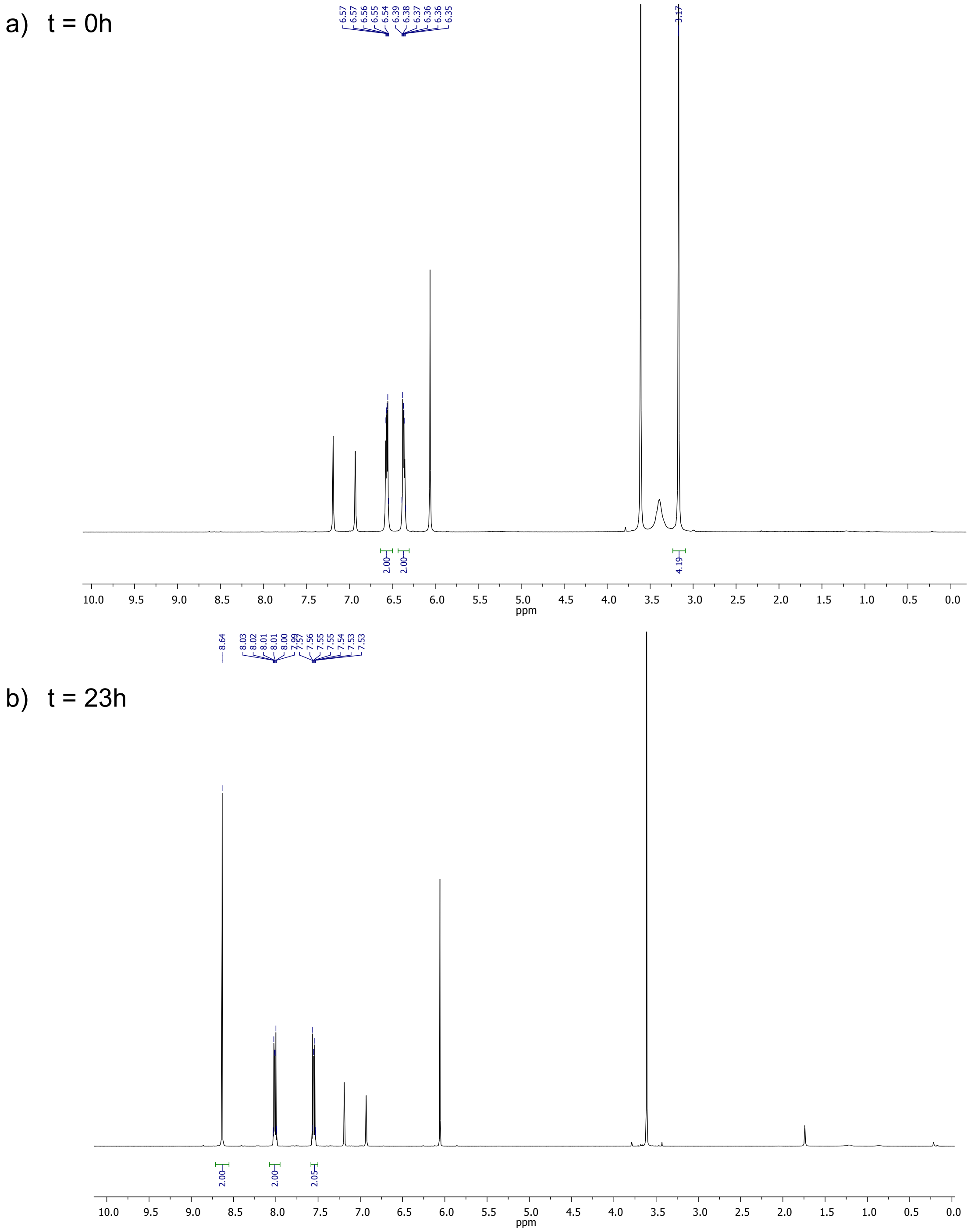

Figure S3 Acceptorless dehydrogenation of **4H**. Initial ^{1}H NMR spectrum (a) and after t = 23 h reaction (b) using 1,3,5-trimethoxybenzene (signals at 3.61 and 6.06 ppm) as an internal standard. Residual *o*-DCB solvent signals at 6.93 and 7.19 ppm.

Selectivity studies in the conversion of 6-Mehtyl-tetrahydroquinoline (**6H**).

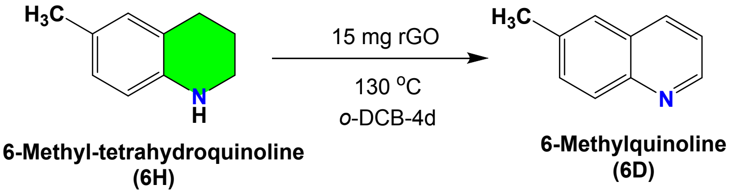

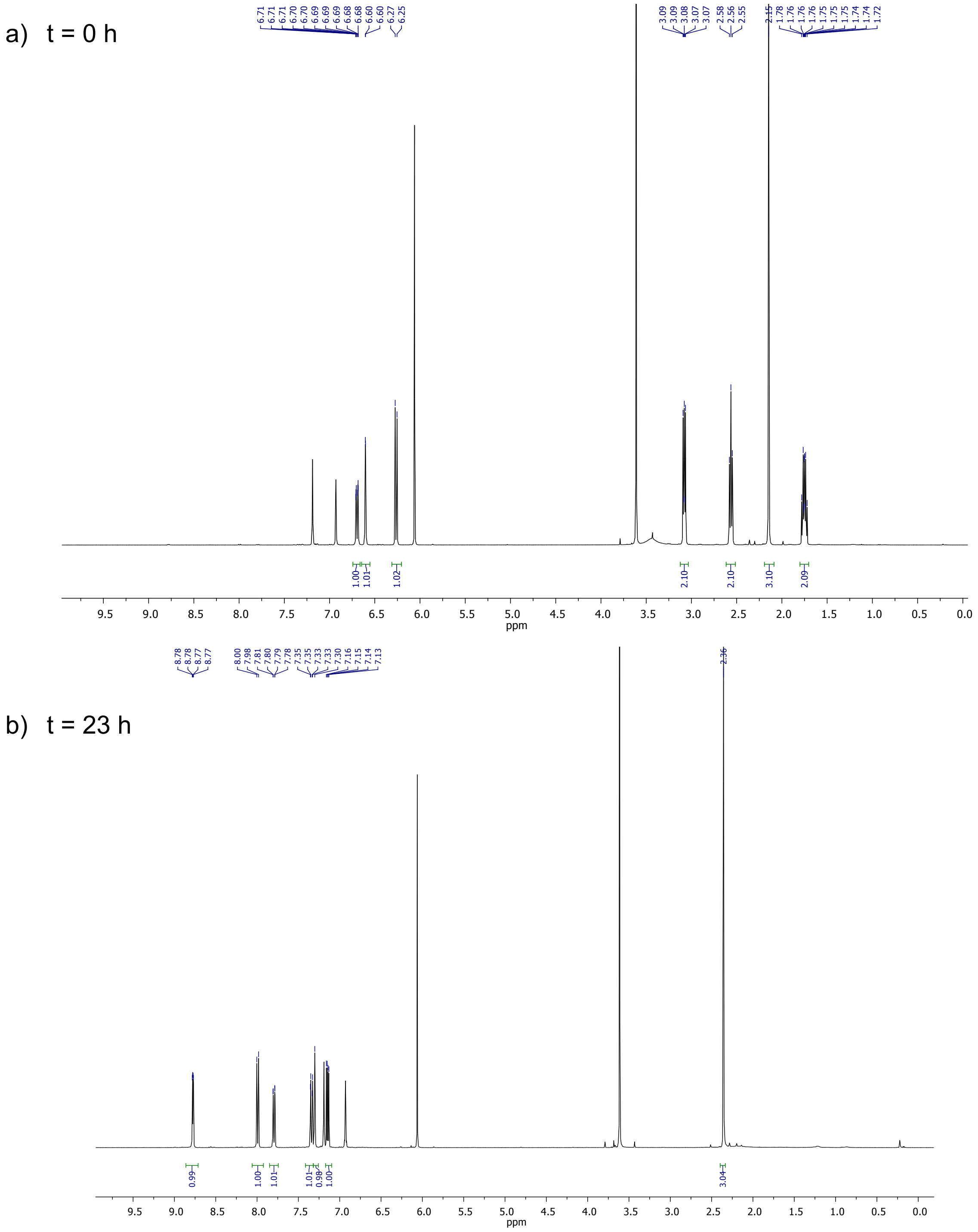

Figure S4 Acceptorless dehydrogenation of **6H**. Initial ^{1}H NMR spectrum (a) and after t = 23 h reaction (b) using 1,3,5-trimethoxybenzene (signals at 3.61 and 6.06 ppm) as an internal standard. Residual *o*-DCB solvent signals at 6.93 and 7.19 ppm.

S6 Scope and limitations: Reaction progress profiles

The scope and limitations of rGO as carbocatalyst were investigated using different N-heterocycles. The reactions were monitored by gas chromatography (GC/FID) using 1,3,5-trimethoxybezene as a standard. In a typical reaction, substrate (0.15 mmol) and rGO (15 mg) were mixed in *o*-DCB (1 mL) at 130 ºC under nitrogen.

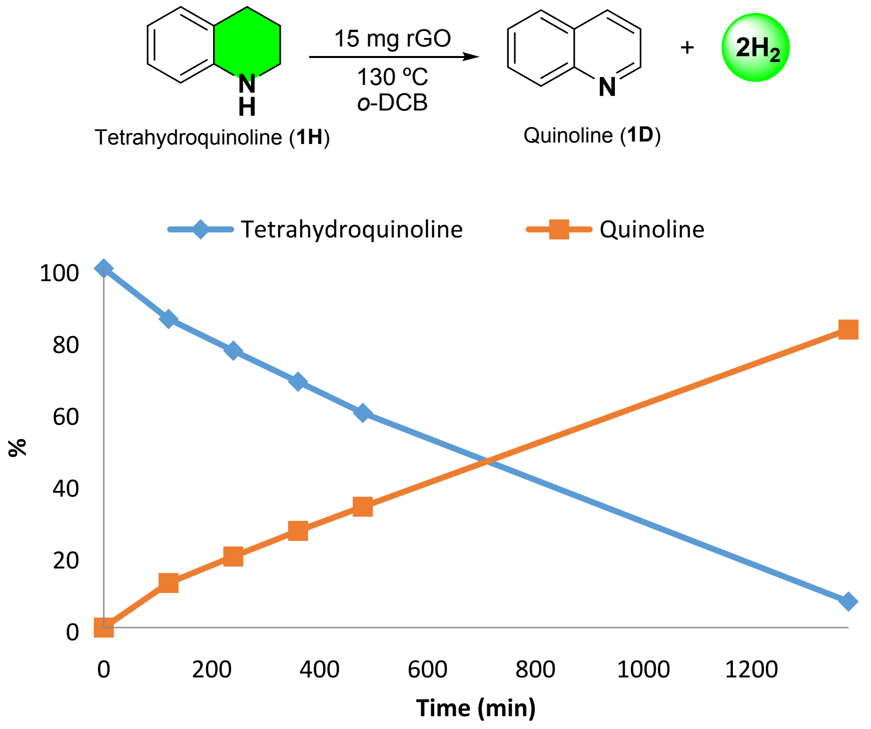

Figure S5 Reaction progress profile in the conversion of **1H** to **1D**. (**H** = hydrogenated and **D** = Dehydrogenated)

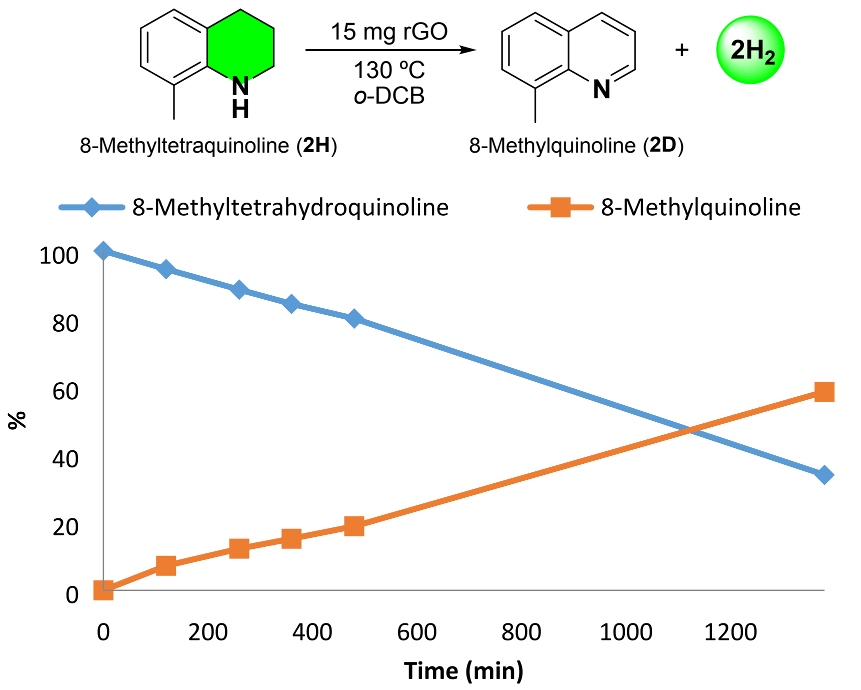

Figure S6 Reaction progress profile in the conversion of **2H** to **2D**.

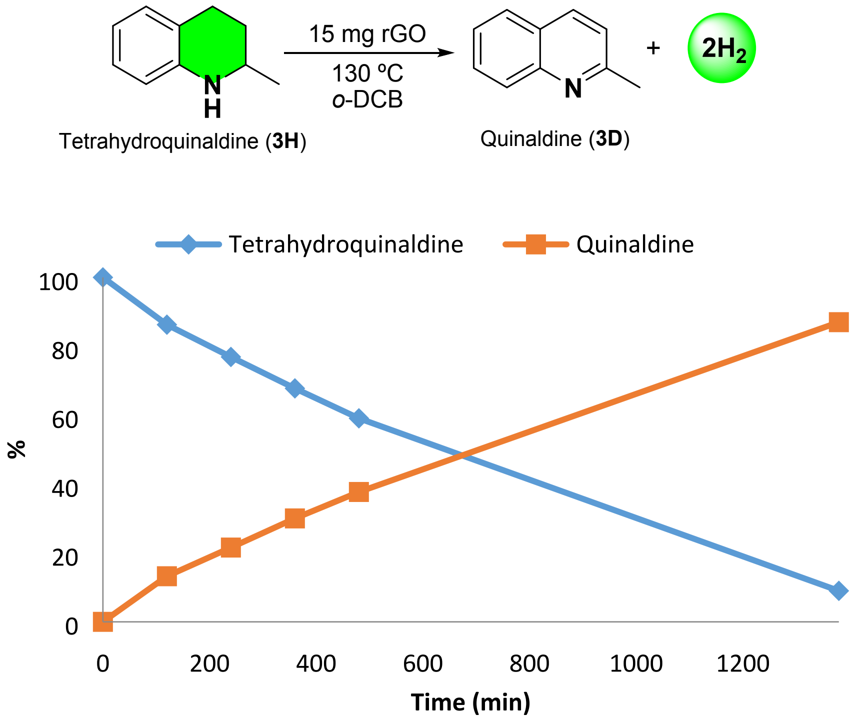

Figure S7 Reaction progress profile in the conversion of **3H** to **3D**.

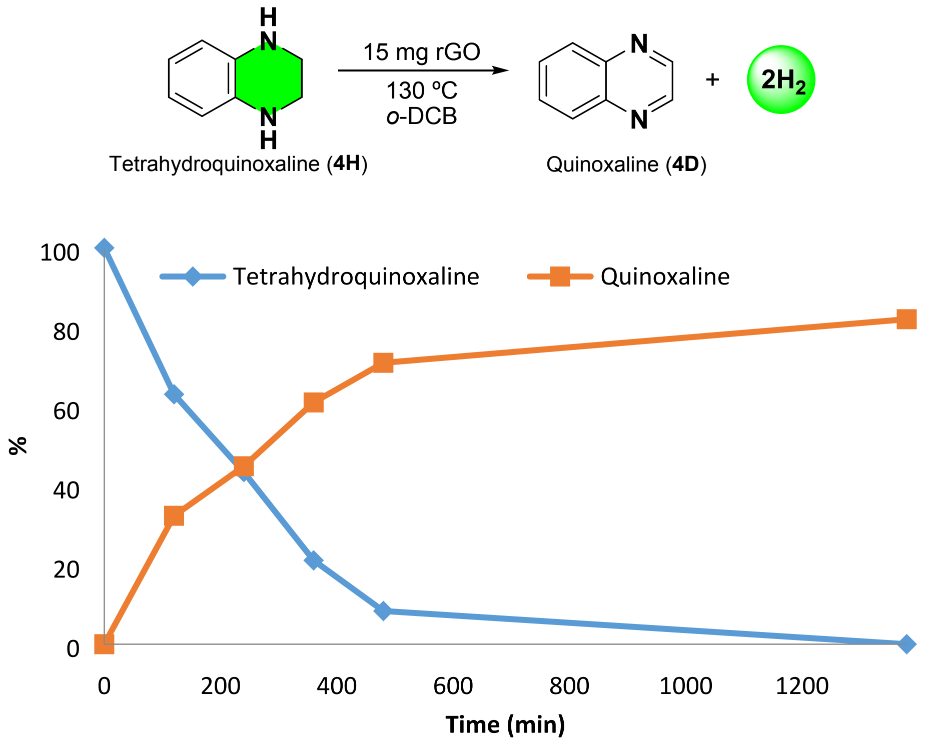

Figure S8 Reaction progress profile in the conversion of **4H** to **4D**.

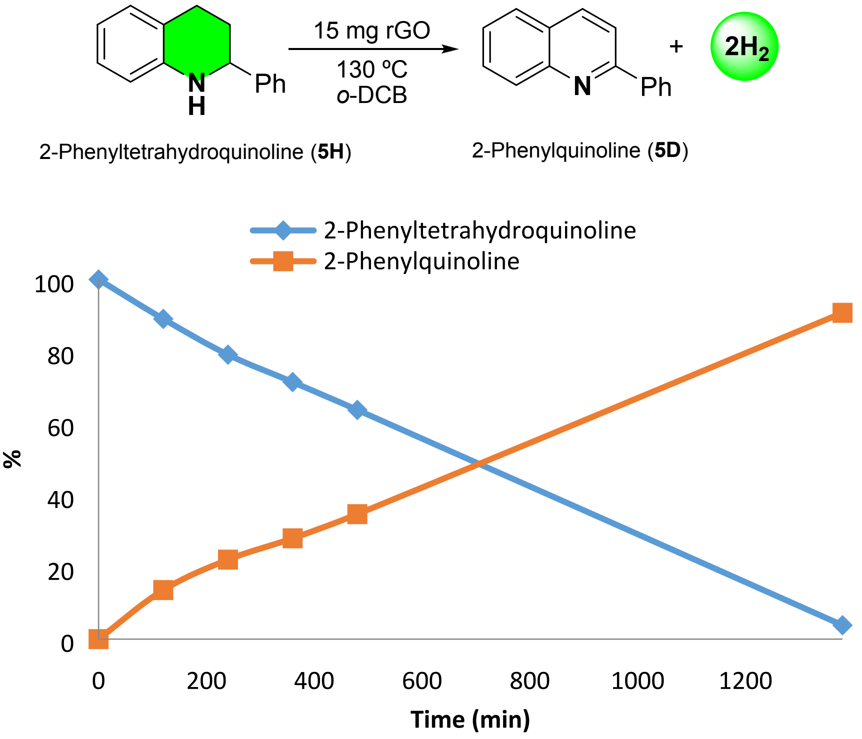

Figure S9 Reaction progress profile in the conversion of **5H** to **5D**.

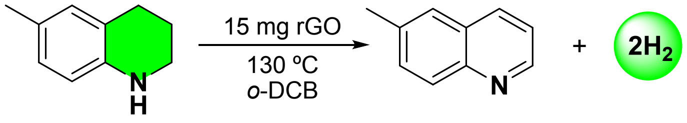

6-Methyltetrahydroquinoline (**6H**) 6-Methylquinoline (**6D**)

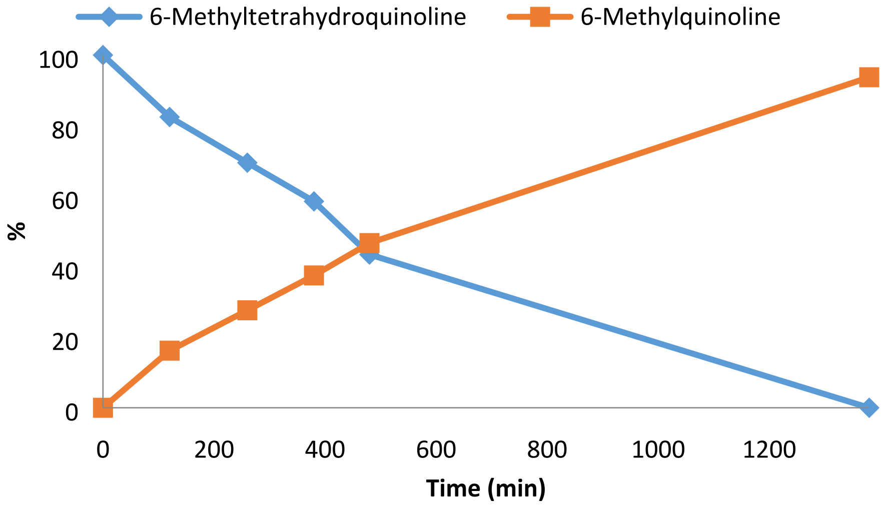

Figure S10 Reaction progress profile in the conversion of **6H** to **6D**.

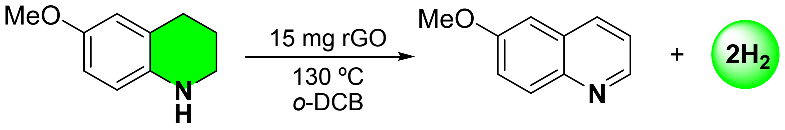

6-Methoxytetrahydroquinoline (**7H**) 6-Methoxyquinoline (**7D**)

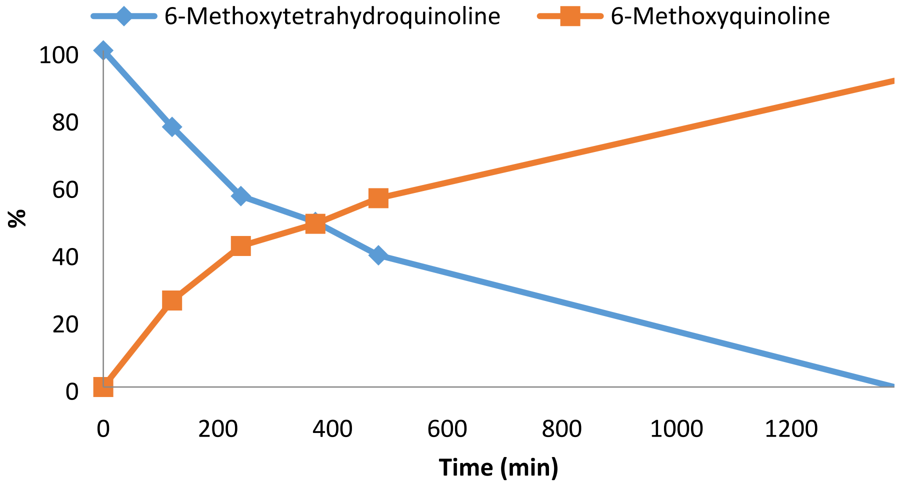

Figure S11 Reaction progress profile in the conversion of **7H** to **7D**.

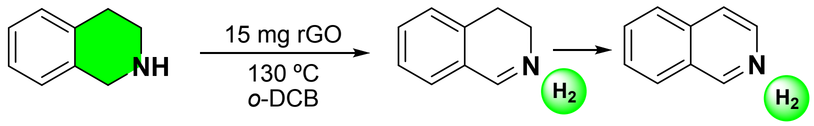

Tetrahydroisoquinoline (**10H**) 3,4-dihydroisoquinoline (**dhiq**) Isoquinoline (**10D**)

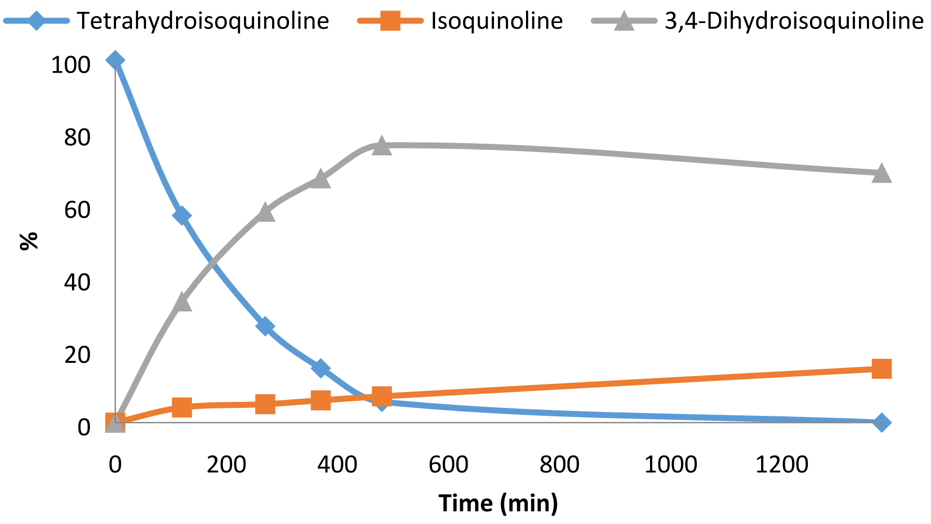

Figure S12 Reaction progress profile in the conversion of **10H** to **10D** and **dhiq**.

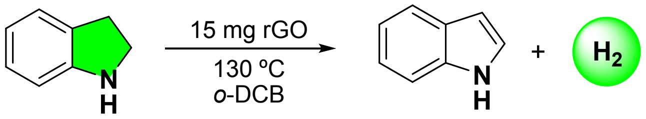

Indoline (**11H**) Indole (**11D**)

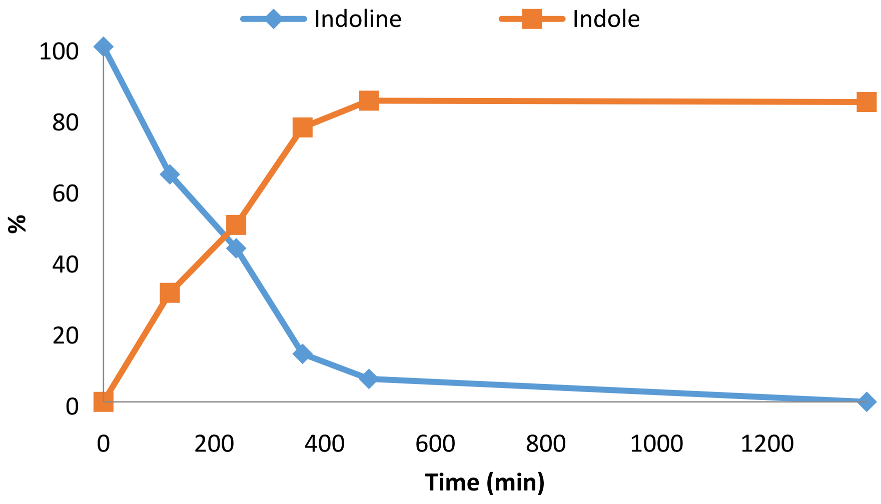

Figure S13 Reaction progress profile in the conversion of **11H** to **11D**.

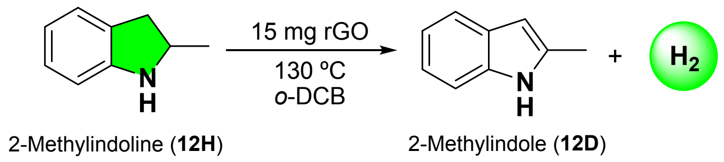

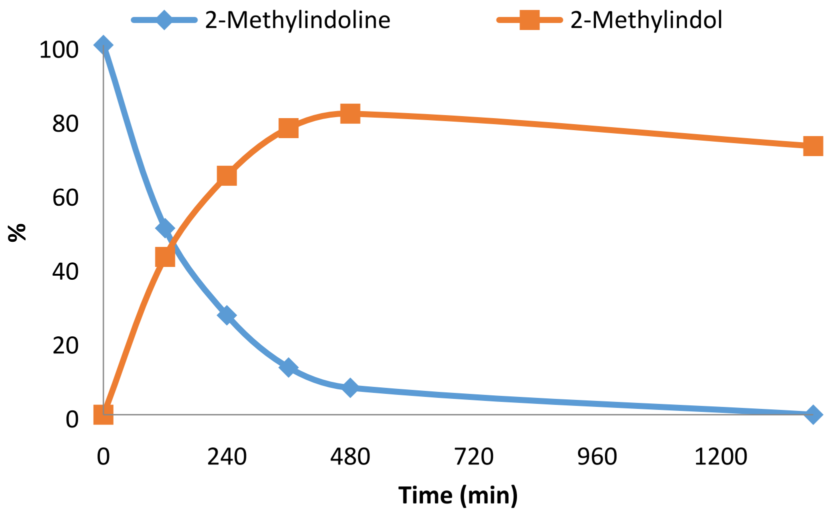

Figure S14 Reaction progress profile in the conversion of 12**H** to 12**D**.

Table S2 Comparison of the initial conversion rates in ADH of N-heterocycles for substrates included in Table 2.

Entry	Substrate	Conversion rate (mmol g_{cat}^{-1} h^{-1})	Entry	Substrate	Conversion rate (mmol g_{cat}^{-1} h^{-1})
1	1H	1.086E-5	7	7H	1.920E-5
2	2H	4.172E-6	8	8H	2.559E-6
3	3H	1.673E-5	9	9H	-
4	4H	2.864E-5	10	10H	3.655E-5
5	5H	8.679E-6	11	11H	2.665E-5
6	6H	1.263E-5	12	12H	4.271E-5

S7 Recycling experiment: Fresh and spent rGO characterization.

HRTEM microscopy

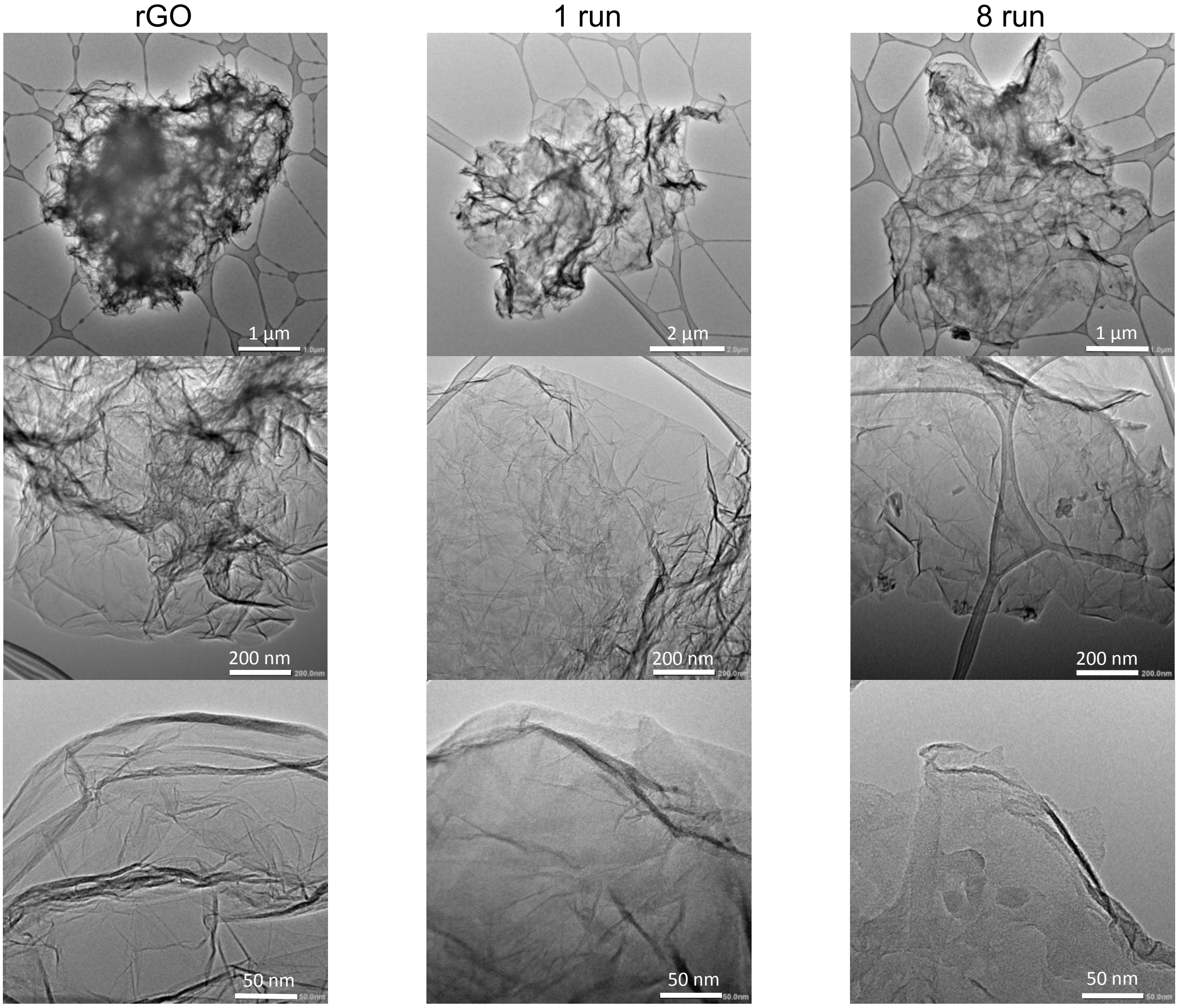

Figure S15 HRTEM images of rGO at different magnifications at selected runs.

X-ray photoelectron spectroscopy (XPS)

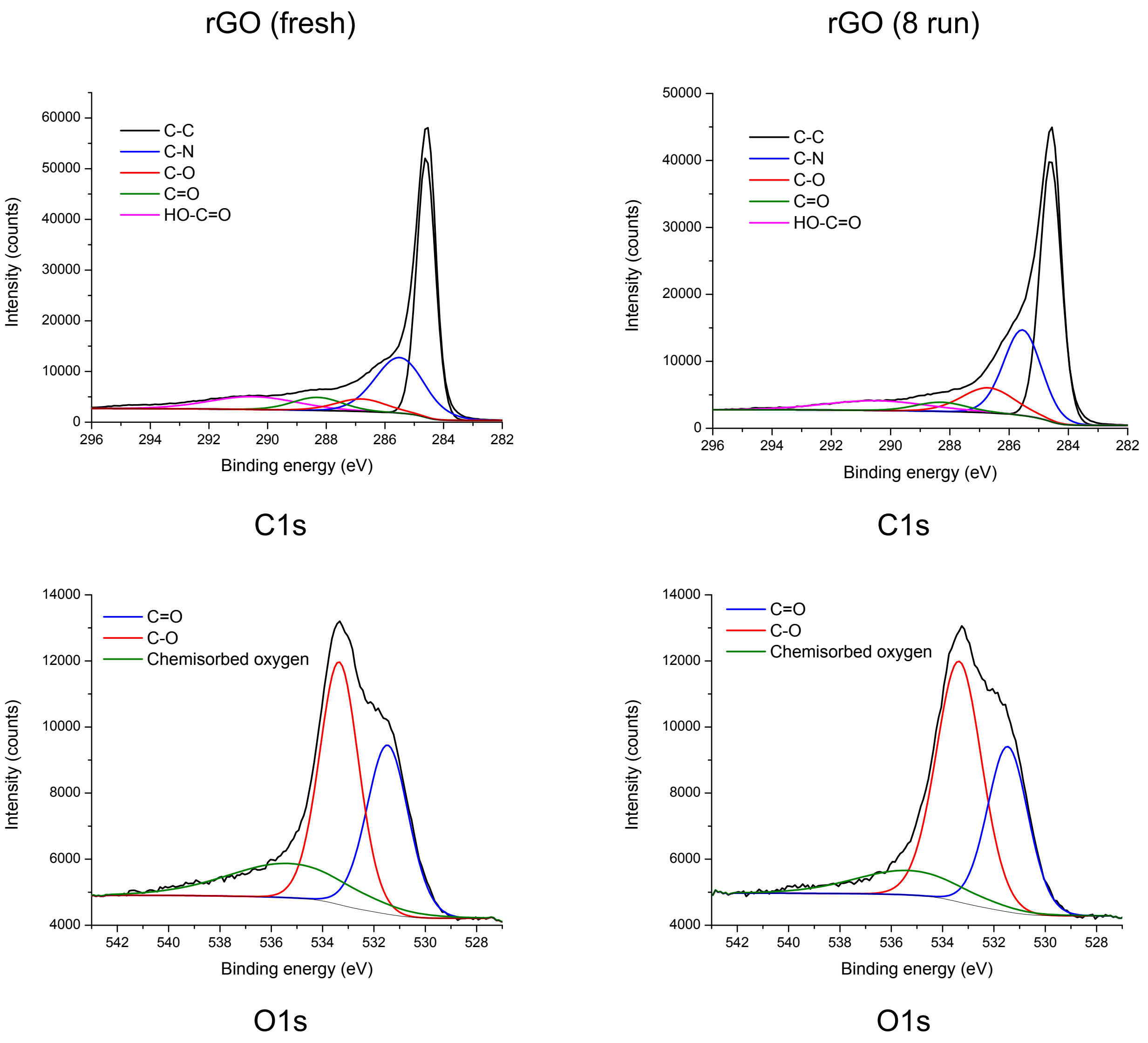

Figure S16 X-ray photoelectron spectroscopy (XPS) characterization of fresh and spent (run 8) rGO showing the binding energies of C1s and O1s.

Combustion Analysis

During the recycling experiment, a sample of the rGO carbocatalysts was obtained at selected times and analyzed by combustion analysis.

Table S3 Combustion analysis of rGO at different runs.

Run	% C	% H	% N	% O	Ratio O/C
0	81.250	0.641	<1	14.350	0.176
1	76.160	1.719	1.405	13.470	0.177
8	73.210	1.991	1.906	14.760	0.202

Raman spectroscopy

Raman spectroscopy is a potential tool in the characterization carbonaceous materials. Raman spectra of rGO were collected at selected runs during the recycling experiment (Figure S7.4). The results reveal the typical graphene pattern with characteristic D, G and 2D bands. The relative intensity (I_D/I_G) of D and G bands is used as an indicator for the extend of defects occurring on graphene materials. The results of the relative intensity (I_D/I_G) during the recycling experiment are maintained up to 8 runs indicating the high stability the rGO.

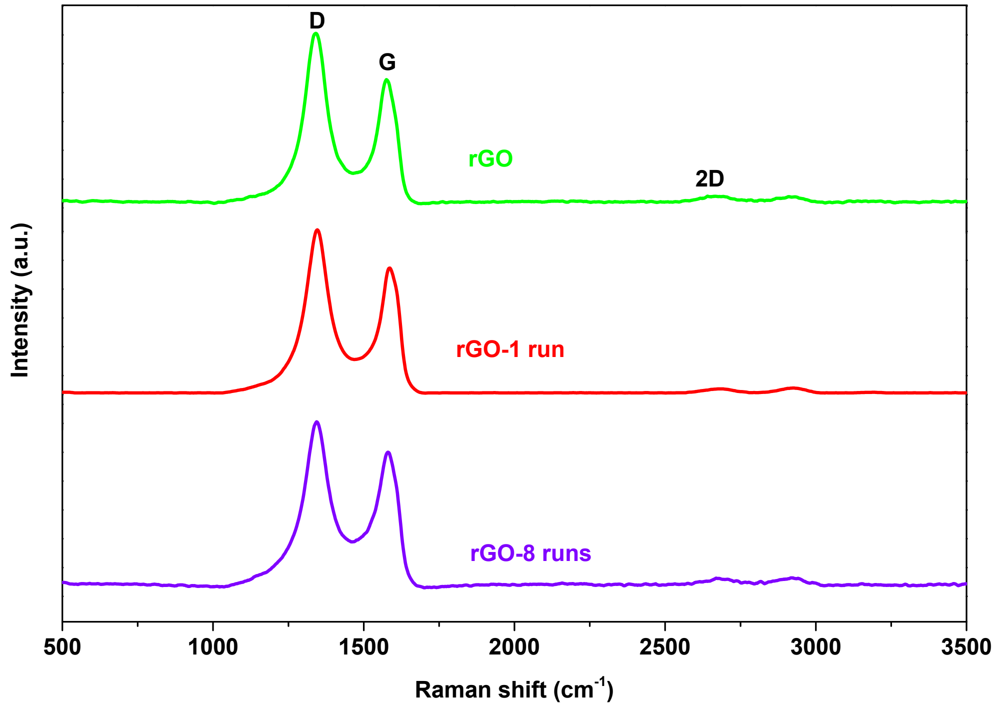

Figure S17 Raman spectra of rGO during the recycling experiment.

S8 Product characterization

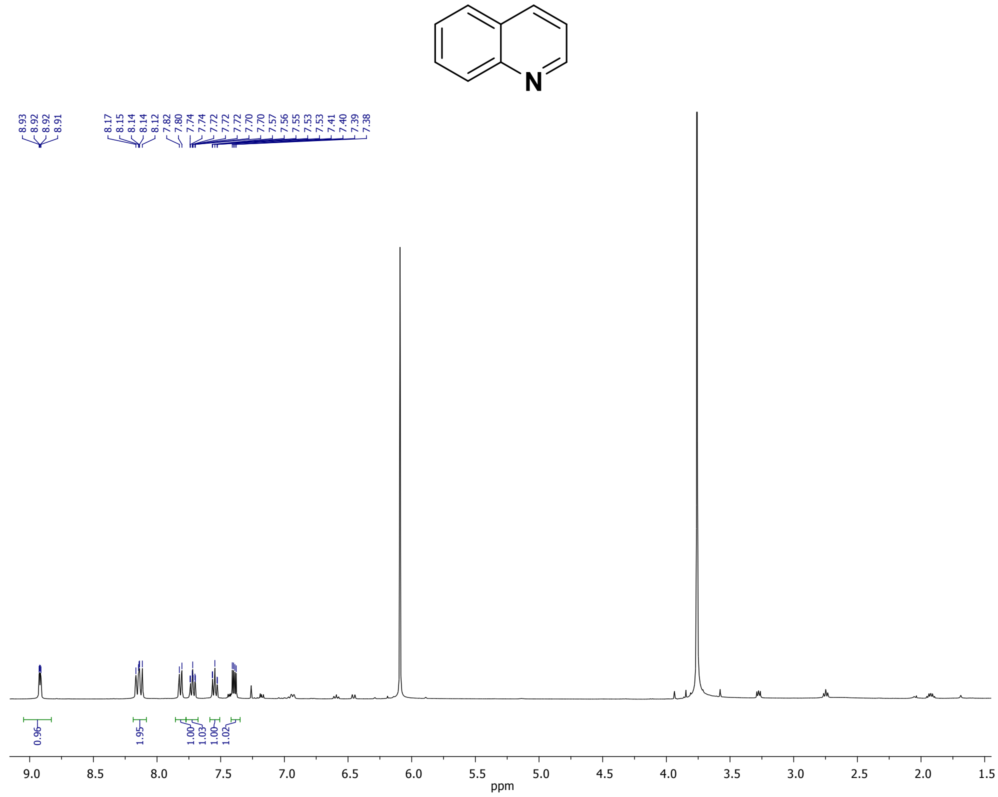

Figure S18 ^{1}H NMR spectrum of Quinoline (**1D**) with 1,3,5-trimethoxybenzene (signals at 3.77 and 6.09 ppm) used as a reference in $CDCl_3$.

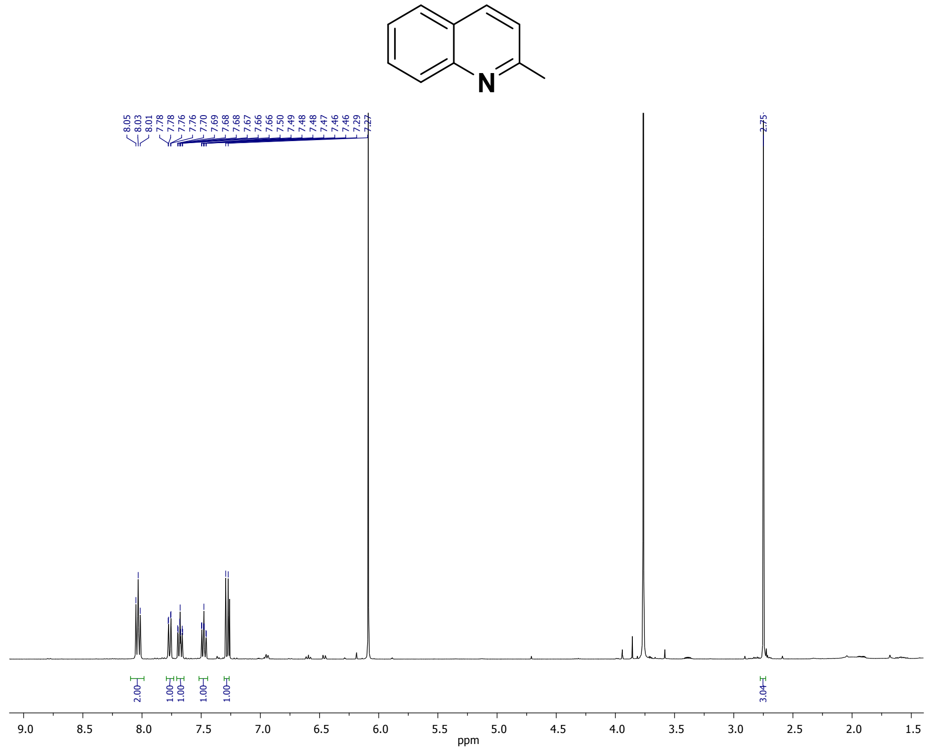

Figure S19 ^{1}H NMR spectrum of Quinaldine (**3D**) with 1,3,5-trimethoxybenzene used as a reference in $CDCl_3$.

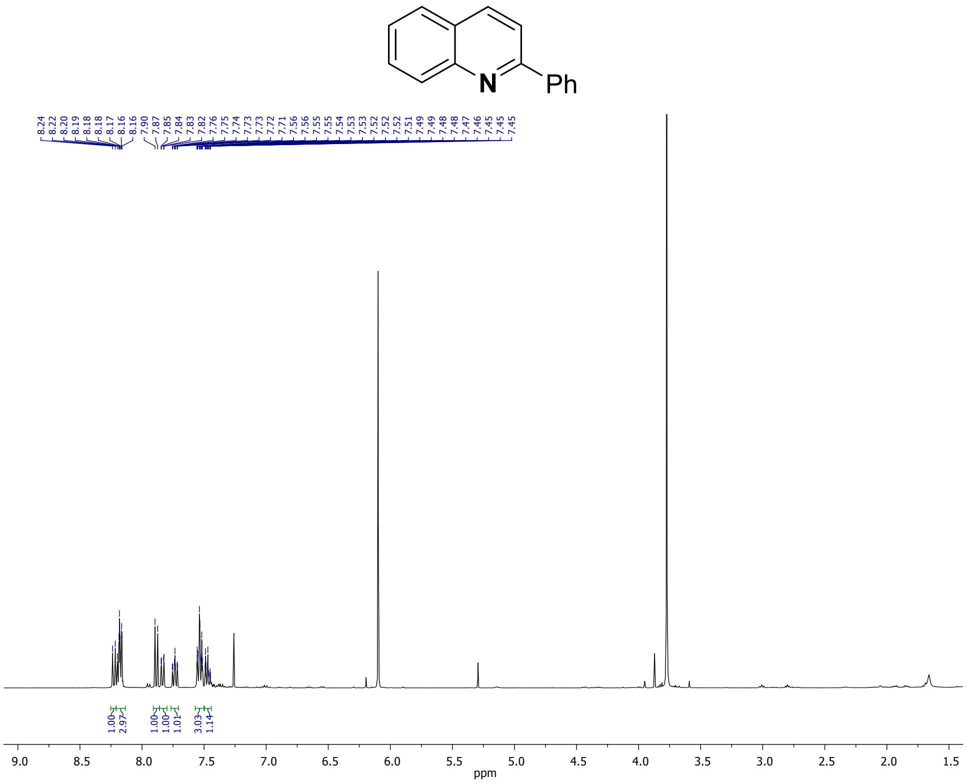

Figure S20 ^{1}H NMR spectrum of 2-Phenylquinoline (**5D**) with 1,3,5-trimethoxybenzene used as a reference in $CDCl_3$.

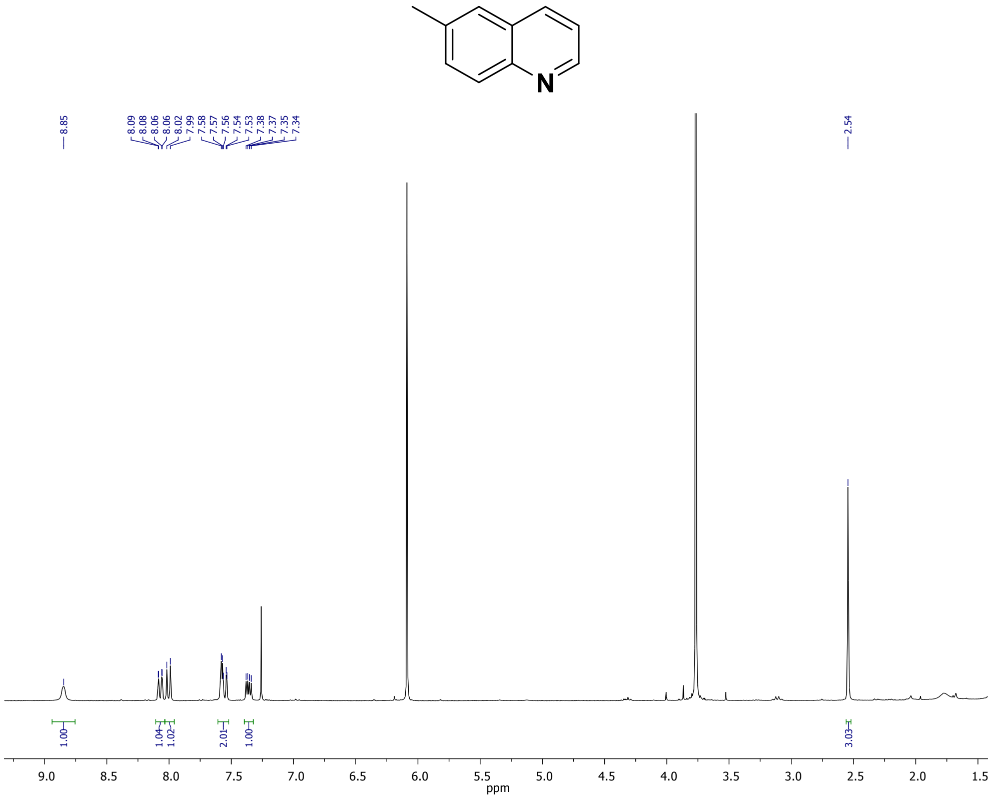

Figure S21 ^{1}H NMR spectrum of 6-Methylquinoline (**6D**) with 1,3,5-trimethoxybenzene used as reference in $CDCl_3$

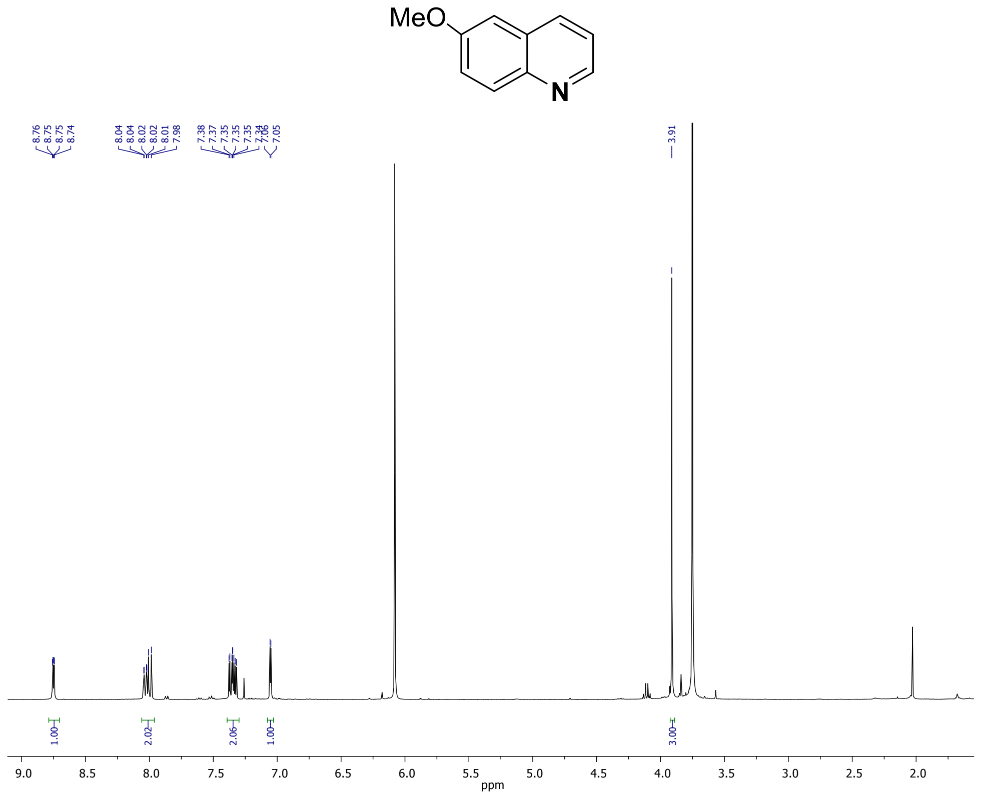

Figure S22 ^{1}H NMR spectrum of 6-Methoxyquinoline (**7D**) with 1,3,5-trimethoxybenzene used as reference in $CDCl_3$.

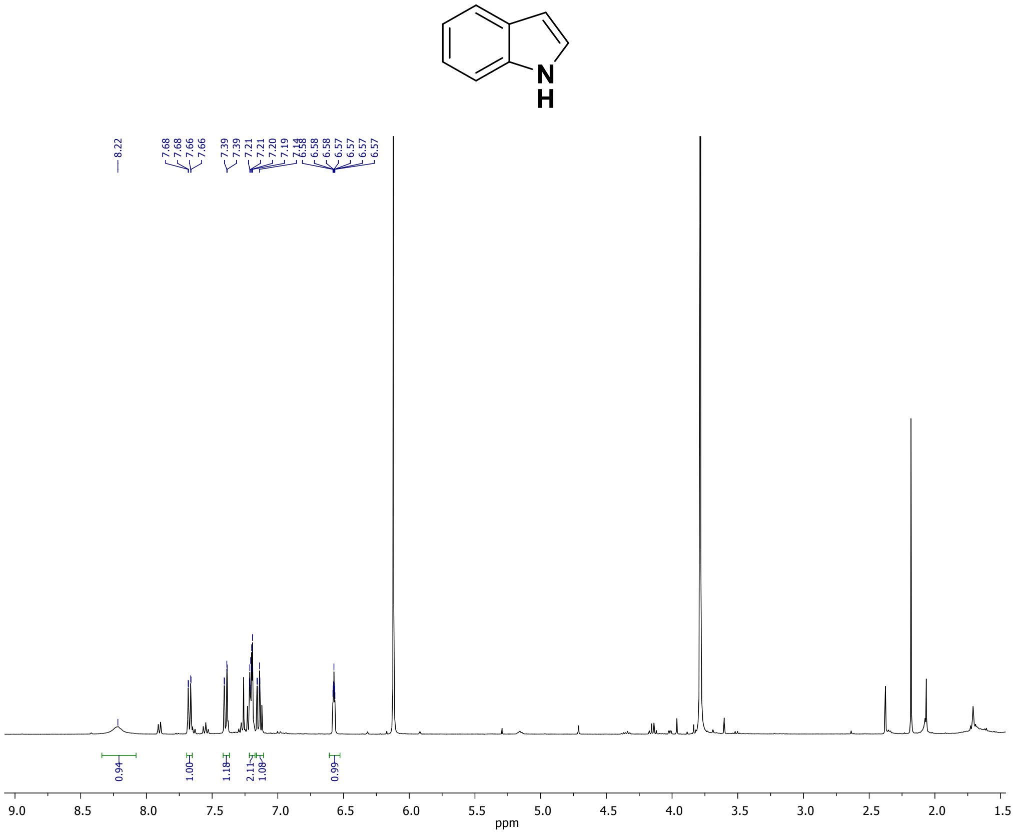

Figure S23 ^{1}H NMR spectrum of Indole (**11D**) with 1,3,5-trimethoxybenzene used as reference in $CDCl_3$.

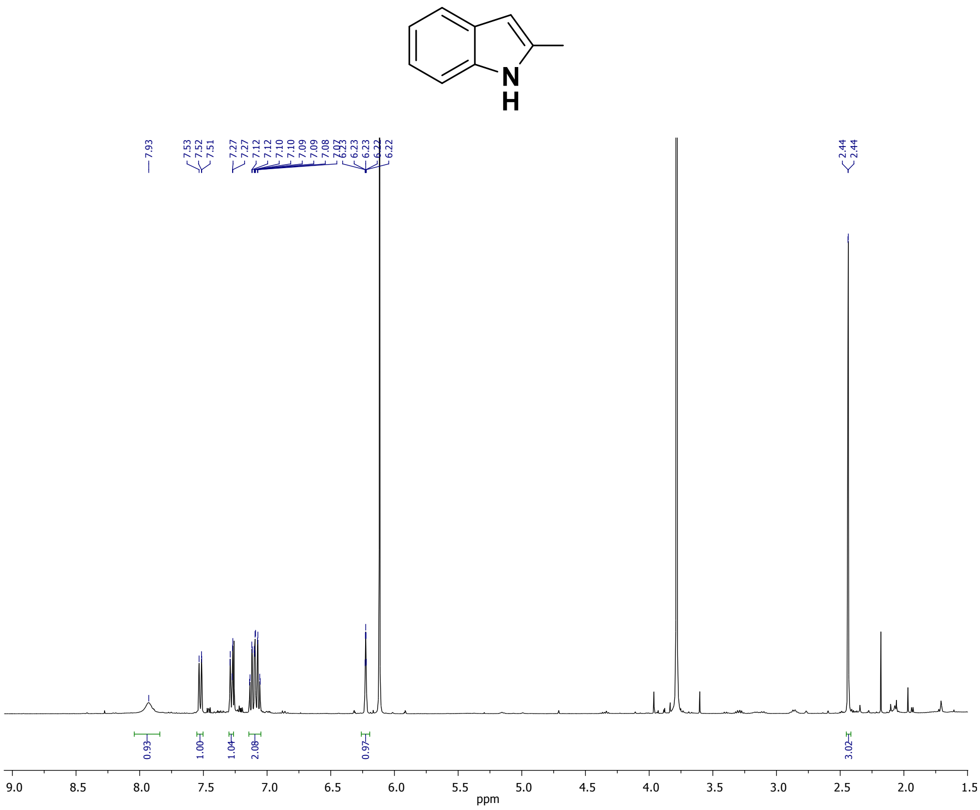

Figure S24 ^{1}H NMR spectrum of 2-Methylindole (**12D**) with 1,3,5-trimethoxybenzene used as reference in $CDCl_3$

S9 Catalytic properties of graphene materials in dehydrogenation of N-heterocycles

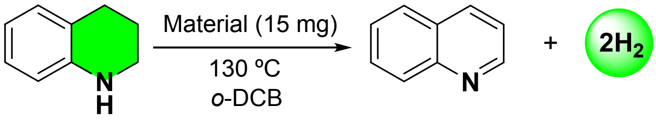

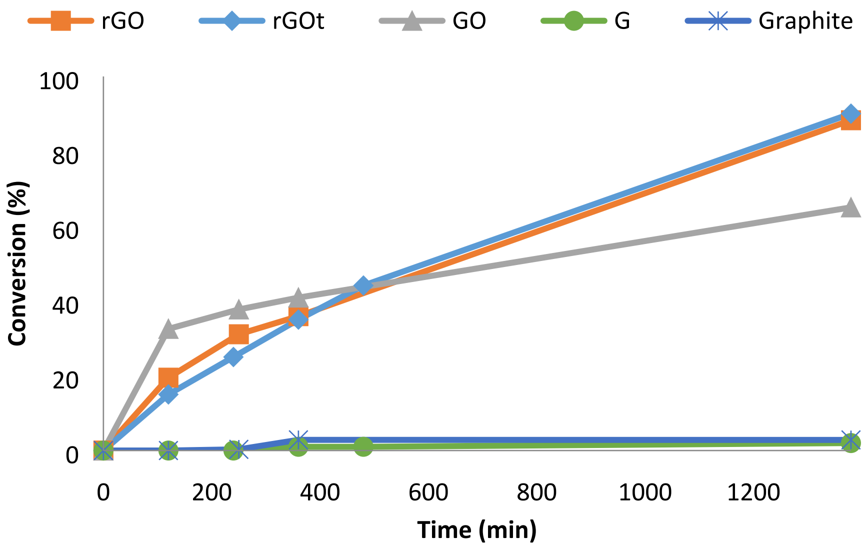

Figure S25 Reaction progress profile in dehydrogenation of tetrahydroquinoleine (**1H**) to quinoline (**1D**) using different graphene-related materials. Conversion obtained by GC/FID using 1,3,5-trimethoxybenzene as an internal standard.

Comparative X-ray photoelectron spectroscopy (XPS) of chemical and thermal rGO.

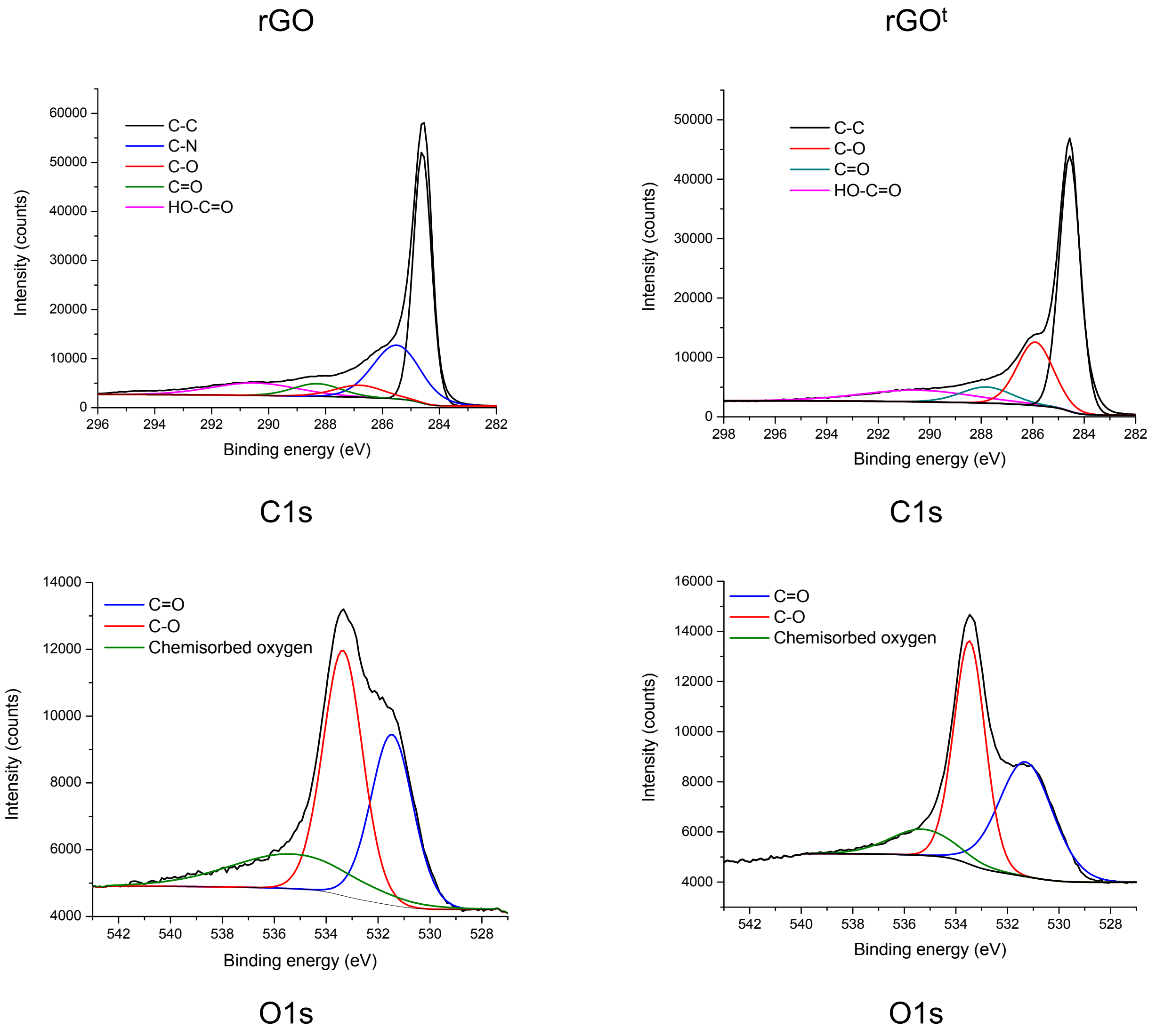

Figure S26 Comparative X-ray photoelectron spectroscopy (XPS) characterization of rGO and rGO^t showing the binding energies of C1s and O1s.

S10 Influence of metal ions in the catalytic properties of rGO

Table S4 ICP/MS analysis of iron and manganese in rGO

Catalyst	Mn (wt%)	Mn (ppm)	Fe(wt%)	Fe (ppm)
rGO	0.2400	2400	0.0420	420

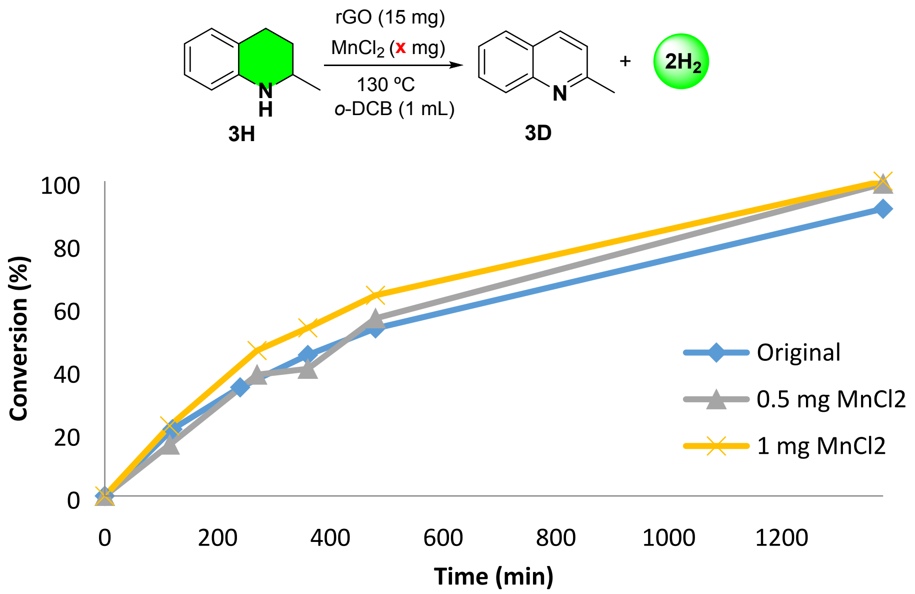

Figure S27 Reaction progress profile in dehydrogenation of **3H** into **3D** under standard conditions with the addition of different amounts of Mn^{2+}. Conversion obtained by GC/FID using 1,3,5-trimethoxybenzene as an internal standard.

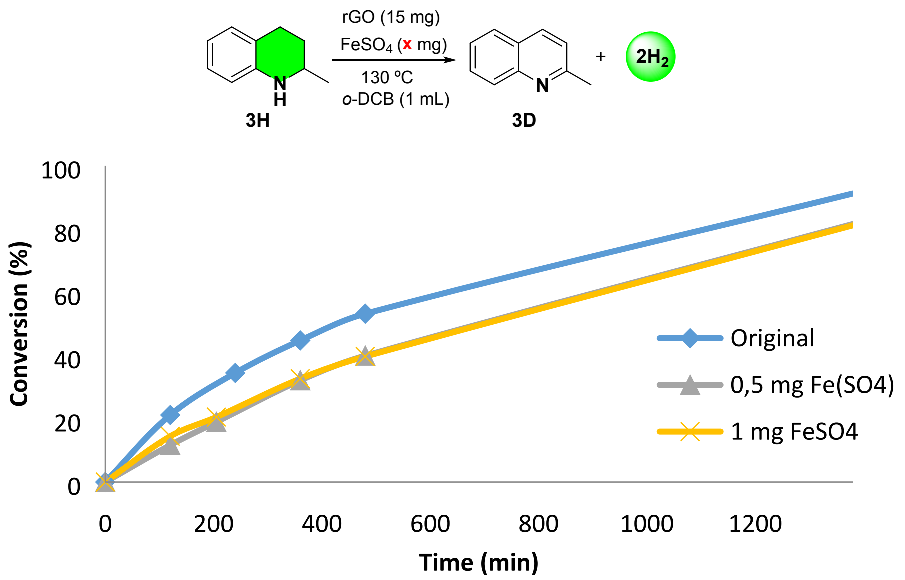

Figure S28 Reaction progress profile in dehydrogenation of **3H** into **3D** under standard conditions with the addition of different amounts of Fe^{2+}. Conversion obtained by GC/FID using 1,3,5-trimethoxybenzene as an internal standard.

S11 Functional groups: Model molecules used as carbocatalyst

Model molecules used as carbocatalysts that mimic the functional groups found in the structure of reduced graphene oxide (**rGO**) including systems with a polyaromatic backbone.

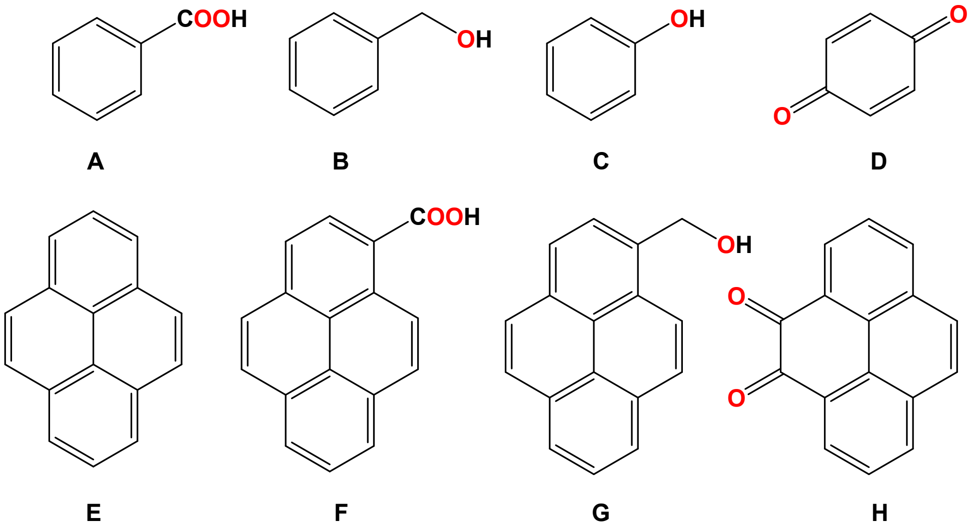

Figure S29 Model molecules used as carbocatalyst for dehydrogenation of N-heterocycles.

Table S5 Catalytic properties of model compounds in dehydrogenation of 2-methyltetrahydroquinoline (**3H**).

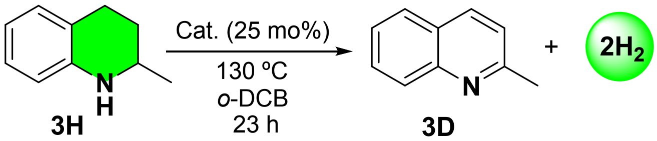

Entry	Cat.	Yield
1	-	<1
2	**A**	19
3	**B**	<1
4	**C**	9
5	**D**	8
6	**E**	<1
7	**F**	9
8	**G**	<1
9	**H**	34

Conditions: Anaerobic conditions using N_2. 2-methyltetrahydroquinoline (0.15 mmol), o-dichlorobenzene (1 mL), T=130 ºC and model molecule (25 mol%). Conversion and yield determined by GC/FID using trimethoxybenzene as an internal standard.

S12 Dehydrogenation sequence of N-heterocycles

Dehydrogenation of N-heterocycles produces two equivalents of molecular hydrogen and is proposed to be stepwise. First dehydrogenation may happen at the NH-C(2)H (imine intermediate) or at the C(3)H-C(4)H remote position. In order to stablish the dehydrogenation sequence procedure, we used a model compound where dehydrogenation next to N is not possible. In this situation dehydrogenation of C(3)H-C(4)H is not produced, suggesting that dehydrogenation sequence in N-heterocycles is produced via dehydrogenation next to N, tautomerization and second dehydrogenation.

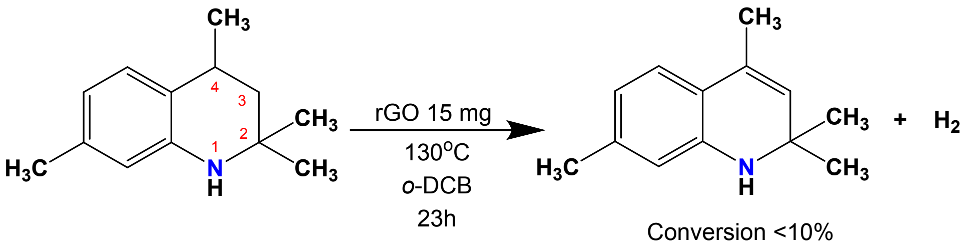

Figure S30 Dehydrogenation of 1,2,3,4-tetrahydro-2,2,4,7-tetramethylquinoline

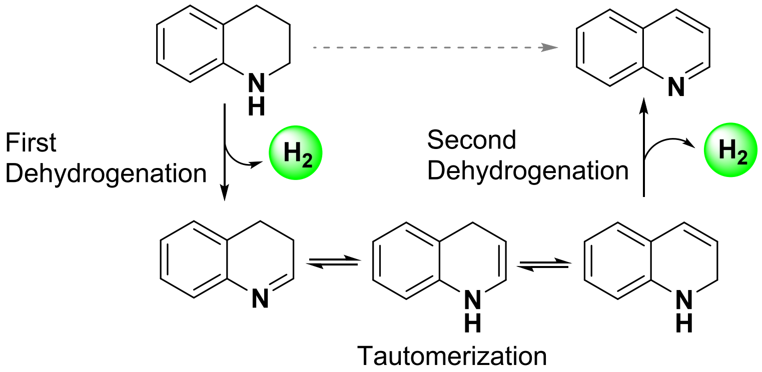

Figure S31 Proposed double dehydrogenation sequence of N-heterocycles

S13 Masking experiments

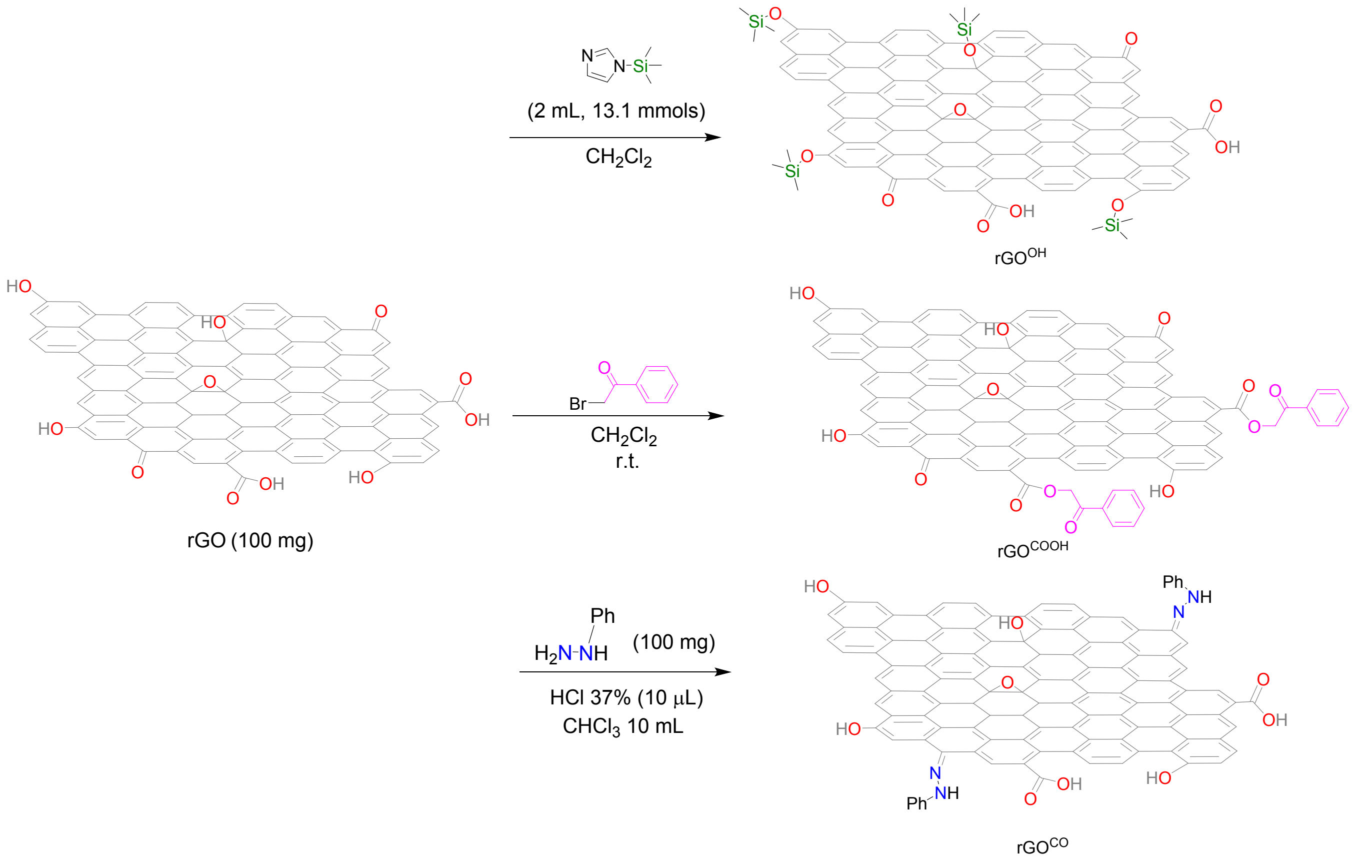

Scheme S1 Synthesis of masked rGO derivatives

Masking of phenol type groups(rGO^{OH})

rGO^{OH} was synthesized by adapting a previously described procedure.[4] rGO (100 mg) was dispersed in 10 mL of dry CH_2Cl_2 and in an ultrasonic bath for 30 min. Then 200 μL of trimethylsilylimidazole (1.31 mmol) was added and the mixture was stirred under N_2 for 12 h at 60 °C. The suspension was filtered out and washed with CH_2Cl_2, water and acetone giving a black solid.

Masking of carboxylic acid groups (rGO^{COOH})

rGO^{COOH} was synthesized adapting a procedure previously described.[5] 2-bromo-1-phenylethanone (200 mg, 1 mmol) and 100 mg rGO were mixed in 5 mL of dry CH_2Cl_2 and stirred at room temperature for 5 h under N_2 in the dark. The mixture was filtered out washed with CH_2Cl_2, water and acetone to remove the physical adsorbed molecules of 2-bromo-1-phenylethanone, giving a black solid.

Masking of ketonic carbonyl groups (rGO^{CO})

rGO^{CO} was synthesized adapting a procedure previously described.[5] Phenylhydrazine (200 mg, 1.81 mmol) and 10 μL HCl acid (37 %) were dissolved in 10 ml of dry CH_2Cl_2.Then, 100 mg rGO was added to the solution and was stirred for 72h under N_2. The mixture was filtered out, washed with CH_2Cl_2, water and acetone to remove the physical adsorbed molecules of phenyl hydrazine, giving a black solid.

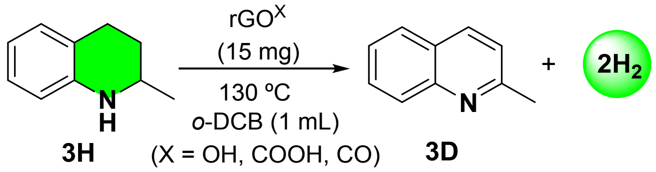

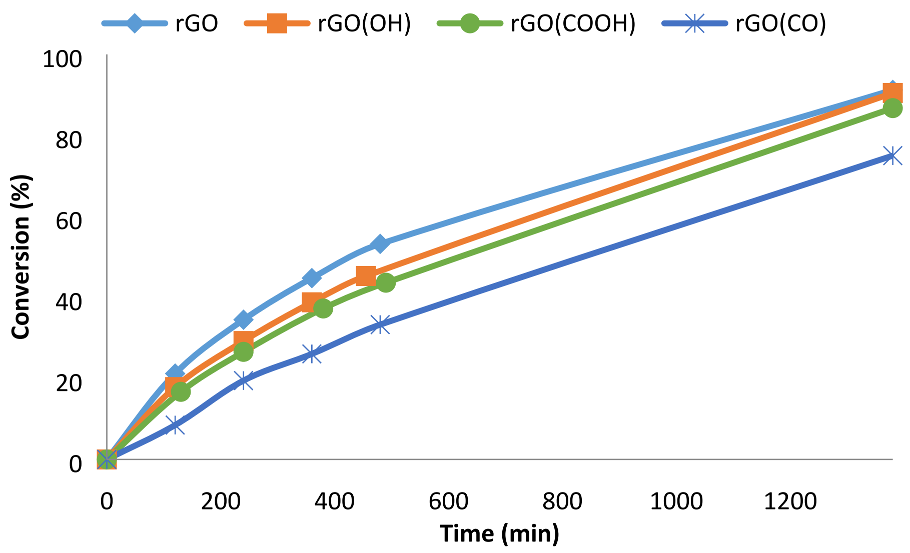

Figure S32 Reaction progress profile in dehydrogenation of **3H** into **3D** under standard conditions using masked rGO derivatives. Conversion obtained by GC/FID using 1,3,5-trimethoxybenzene as an internal standard.

S14 Reported catalysts comparison in dehydrogenation of tetrahydroquinaldine dehydrogenation.

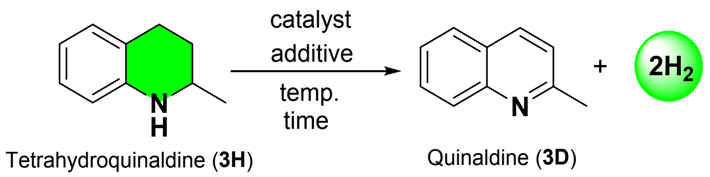

Entry	Catalyst	Loading	Solvent	Additive	T[a] (°C)	t (h)	Yield	Ref.
1	rGO	15 mg (19.6 mol%)*	*o*-DCB	-	130	23	87	This work
2	rGO	5 mg (6.5 mol%)*	*o*-DCB	-	130	24	71	This work
3	PdNPs/SBA-15	1 mol%	-	-	130	23	24	[6]
4	RhNPs/SBA-15	1 mol%	-	-	130	23	60	[6]
5	PtNPs/SBA-15	1 mol%	-	-	130	23	100	[6]
6	$RhCl_3$	5 mol%	-	-	130	23	26	[6]
7	$Rh(acac)_3$	0.5 mol%	-	-	130	23	4	[6]
8	K_2PtCl_4	0.5 mol%	-	-	130	23	6	[6]
9	Pt/C	0.5 mol%	Toluene	-	130	23	8	[6]
10	K_2PdCl_4	5 mol%	-	-	130	23	15	[6]
11	$PdCl_2$	0.5 mol%	-	-	130	23	8	[6]
12	Co-Phen@C	6 mol%	*n*-Decane	*t*-BuOK	150	36	94	[7]
13	Pt/C	0.1 mol%	*o*-xylene	-	145	60	93	[8]
14	tBuOK	100 mol%	*o*-xylene	-	140	36	66	[9]
15	$NiNPs/SiO_2$	20 mol %	triglyme	-	200	24	65	[10]
16	Cu/TiO_2	20 mol%	mesitylene	-	150	16	99	[11]
17	ISAS-Co/OPNC	1.5 mol%	mesitylene	-	120	8	96	[12]
18	Cp*Ir complex	2.0 mol%	*p*-xylene	-	110	20	100	[13]

*Catalyst loading expresed in mol% considering the C=O groups as active sites. This estimation is obtained by considering the total amount of oxygen obtained from elemental analysis and the percentage of the oxygen corresponding to C=O groups obtained from XPS. The catalyst loading is in the range of molecular organocatalysts.

S15 References

(1) Hummers, W. S.; Offeman, R. E. Preparation of Graphitic Oxide. *J. Am. Chem. Soc.* **1958**, *80* (6), 1339.

(2) Espinosa, J. C.; Álvaro, M.; Dhakshinamoorthy, A.; Navalón, S.; García, H. Engineering Active Sites in Reduced Graphene Oxide: Tuning the Catalytic Activity for Aerobic Oxidation. *ACS Sustain. Chem. Eng.* **2019**, *7* (19), 15948–15956. https://doi.org/10.1021/acssuschemeng.9b02237.

(3) Trandafir, M.-M.; Florea, M.; Neaţu, F.; Primo, A.; Parvulescu, V. I.; García, H. Graphene from Alginate Pyrolysis as a Metal-Free Catalyst for Hydrogenation of Nitro Compounds. *ChemSusChem* **2016**, *9* (13), 1565–1569. https://doi.org/10.1002/cssc.201600197.

(4) Sánchez-Page, B.; Jiménez, M. V.; Pérez-Torrente, J. J.; Passarelli, V.; Blasco, J.; Subias, G.; Granda, M.; Álvarez, P. Hybrid Catalysts Comprised of Graphene Modified with Rhodium-Based N-Heterocyclic Carbenes for Alkyne Hydrosilylation. *ACS Appl. Nano Mater.* **2020**, *3* (2), 1640–1655. https://doi.org/10.1021/acsanm.9b02398.

(5) Qi, W.; Liu, W.; Zhang, B.; Gu, X.; Guo, X.; Su, D. Oxidative Dehydrogenation on Nanocarbon: Identification and Quantification of Active Sites by Chemical Titration. *Angew. Chemie Int. Ed.* **2013**, *52* (52), 14224–14228. https://doi.org/10.1002/anie.201306825.

(6) Deraedt, C.; Ye, R.; Ralston, W. T.; Toste, F. D.; Somorjai, G. A. Dendrimer-Stabilized Metal Nanoparticles as Efficient Catalysts for Reversible Dehydrogenation/Hydrogenation of N-Heterocycles. *J. Am. Chem. Soc.* **2017**, *139* (49), 18084–18092. https://doi.org/10.1021/jacs.7b10768.

(7) Jaiswal, G.; Subaramanian, M.; Sahoo, M. K.; Balaraman, E. A Reusable Cobalt Catalyst for Reversible Acceptorless Dehydrogenation and Hydrogenation of N-Heterocycles. *ChemCatChem* **2019**, *11* (10), 2449–2457. https://doi.org/10.1002/cctc.201900367.

(8) Moromi, S. K.; Siddiki, S. M. A. H.; Kon, K.; Toyao, T.; Shimizu, K. Acceptorless Dehydrogenation of N -Heterocycles by Supported Pt Catalysts. *Catal. Today* **2017**, *281*, 507–511. https://doi.org/10.1016/j.cattod.2016.06.027.

(9) Liu, T.; Wu, K.; Wang, L.; Yu, Z. Potassium Tert -Butoxide-Promoted Acceptorless Dehydrogenation of N-Heterocycles. *Adv. Synth. Catal.* **2019**, *361* (17), 3958–3964. https://doi.org/10.1002/adsc.201900499.

(10) Ryabchuk, P.; Agapova, A.; Kreyenschulte, C.; Lund, H.; Junge, H.; Junge, K.; Beller, M. Heterogeneous Nickel-Catalysed Reversible, Acceptorless Dehydrogenation of N-Heterocycles for Hydrogen Storage. *Chem. Commun.* **2019**, *55* (34), 4969–4972. https://doi.org/10.1039/C9CC00918C.

(11) Kaneda, K.; Mikami, Y.; Mitsudome, T.; Mizugaki, T.; Jitsukawa, K. Reversible Dehydrogenation-Hydrogenation of Tetrahydroquinoline-Quinoline Using a Supported Cooper Nanoparticle Catalyst. *Heterocycles* **2010**, *82* (2), 1371. https://doi.org/10.3987/COM-10-S(E)90.

(12) Han, Y.; Wang, Z.; Xu, R.; Zhang, W.; Chen, W.; Zheng, L.; Zhang, J.; Luo, J.; Wu, K.; Zhu, Y.; Chen, C.; Peng, Q.; Liu, Q.; Hu, P.; Wang, D.; Li, Y. Ordered Porous Nitrogen-Doped Carbon Matrix with Atomically Dispersed Cobalt Sites as an Efficient Catalyst for Dehydrogenation and Transfer Hydrogenation of N-Heterocycles. *Angew. Chemie Int. Ed.* **2018**, *57* (35), 11262–11266. https://doi.org/10.1002/anie.201805467.

(13) Yamaguchi, R.; Ikeda, C.; Takahashi, Y.; Fujita, K. Homogeneous Catalytic System for Reversible Dehydrogenation−Hydrogenation Reactions of Nitrogen Heterocycles with Reversible Interconversion of Catalytic Species. *J. Am. Chem. Soc.* **2009**, *131* (24), 8410–8412. https://doi.org/10.1021/ja9022623.
